# NMR‐Based Structural Analysis of Highly Substituted Pyridines From Kondrat'eva Aza‐Diels–Alder Cycloadditions

**DOI:** 10.1002/mrc.70075

**Published:** 2025-12-18

**Authors:** Galdina V. Suárez‐Moreno, Francisco Méndez, Atilano Gutierrez‐Carrillo, Mónica A. Rincón‐Guevara, Yoarhy A. Amador‐Sánchez, Alejandro Islas‐Jácome, Eduardo González‐Zamora

**Affiliations:** ^1^ Instituto Politécnico Nacional Unidad Profesional Interdisciplinaria de Biotecnología Ciudad de México Mexico; ^2^ Departamento de Química Universidad Autónoma Metropolitana‐Iztapalapa Ciudad de México Iztapalapa Mexico; ^3^ Laboratorio Divisional de Espectrometría de Masas, División de Ciencias Biológicas y de la Salud Universidad Autónoma Metropolitana, Unidad Iztapalapa Iztapalapa Ciudad de México Mexico

**Keywords:** ^13^C, ^15^N, ^1^H, Diels–Alder, NMR, pyridines

## Abstract

Pyridines are a crucial class of heterocycles with widespread applications in natural products, pharmaceuticals, and fluorescent organic materials. In this manuscript, we report the results from a kinetic and mechanistic investigation of an inverse‐electron‐demand Diels–Alder (IEDDA) cycloaddition involving an oxazole‐type diene synthesized via an Ugi–Zhu multicomponent reaction (UZ‐3CR). This heterodiene reacts efficiently with various dienophiles such as *E*‐4‐oxopentenoic acid, fumaric acid, and monoethyl maleate, yielding highly substituted pyridines in good to excellent yields. Reaction conditions were optimized, and the influence of solvent polarity on regioselectivity was evaluated. The necessity of protonation for successful cycloadditions was probed using structurally diverse dienophiles, revealing the essential role of the carboxylic acid group in triggering the reactions. Mechanistic insights were supported by a comprehensive NMR study (^1^H, ^13^C, and ^15^N), which provided indirect evidence of in situ protonation of the oxazole ring. Notably, ^15^N NMR revealed significant downfield shifts of the oxazole nitrogen, consistent with its protonation, and the emergence of new nitrogen signals corresponding to pyridine products. This study demonstrates the synthetic utility of Ugi–Zhu‐derived 5‐aminooxazoles in IEDDA cycloadditions and highlights the critical role of acid‐promoted activation in enabling efficient pyridine synthesis. We report the results from a kinetic and mechanistic investigation of an IEDDA cycloaddition involving an oxazole‐type diene synthesized via an UZ‐3CR.

## Graphical abstract

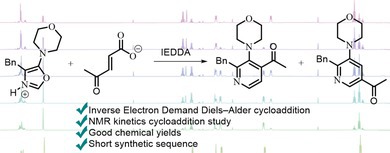

## 1 Introduction

Pyridines are a common heterocyclic scaffold found in numerous natural products [1], bioactive compounds [2], and organic fluorescent materials [3]. Due to their significance, the development of efficient synthetic methodologies to construct this highly valuable heterocyclic motif remains a major objective within the organic synthesis community. Several synthetic strategies have been reported in the literature, including condensation reactions of 1,5‐dicarbonyl compounds with ammonia (Kröhnke pyridine synthesis) [4, 5] or hydroxylamine [6, 7], multicomponent approaches followed by oxidation steps, such as the Hantzsch pyridine synthesis [8, 9, 10], and electrocyclizations of polyunsaturated imines or oximes [11, 12]. Additionally, Diels–Alder (DA) reactions involving 1‐azadienes, isoxazoles, pyrimidines, pyrazines, and 1,2,4‐triazenes have also been employed for pyridine synthesis [13]. Among these methods, DA reactions are particularly effective for constructing highly substituted pyridines. The regiochemical and stereochemical outcome of DA cycloadditions can be influenced by molecular orbital interactions: In normal‐electron‐demand DA reactions, the process is governed by the HOMO of the diene and the LUMO of the dienophile, typically involving electron‐rich dienes and electron‐deficient dienophiles [14]. In contrast, inverse‐electron‐demand DA (IEDDA) reactions are dominated by the interaction between the HOMO of the dienophile and the LUMO of the diene [15]. Notably, the IEDDA reaction is one of the most widely used strategies for pyridine synthesis due to its efficiency in assembling complex substituted derivatives through a Kondrat'eva DA approach [16]. As demonstrated through theoretical studies, the activation of oxazole rings by protonation or the incorporation of electron‐withdrawing groups significantly enhances their reactivity in IEDDA reactions, lowering activation barriers and favoring the formation of pyridine derivatives under mild conditions [17]. Motivated by these findings, we present an NMR‐based kinetic investigation of a Kondrat'eva‐type IEDDA reaction employing a highly substituted oxazole as the diene. The oxazole was synthesized via an Ugi–Zhu three‐component reaction (UZ‐3CR) [18]. It was subsequently reacted with different dienophiles like *E*‐4‐oxopentenoic acid, fumaric acid, and monoethyl maleate to afford highly substituted pyridines in good yields. The reaction kinetics were monitored using nuclear magnetic resonance (NMR) spectroscopy, providing mechanistic insights and evaluating the efficiency of this transformation.

## 2 Results and Discussion

### 2.1 Organic Synthesis

**SCHEME 1:**
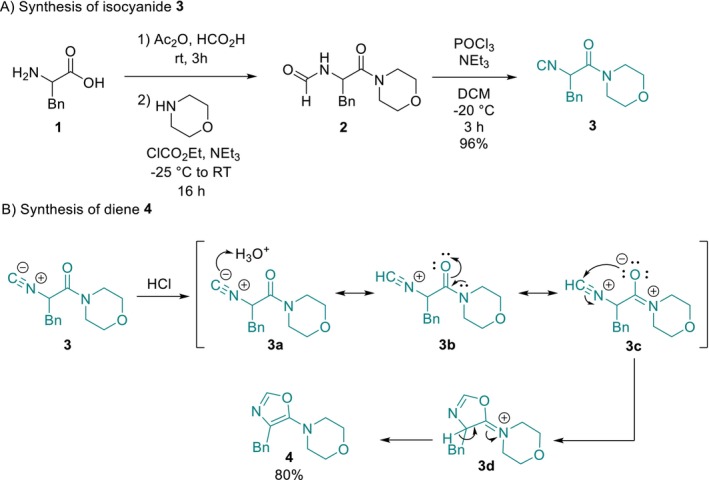
Synthetic protocol toward the synthesis of diene **4**.

We began the study by synthesizing the key isocyanide **3**, which plays a pivotal role in the IEDDA sequence (Scheme 1A). Starting from commercially available phenylalanine (**1**), a formylation step followed by peptide coupling with morpholine afforded the desired formamide **2** in 94% overall yield over two steps. An Ugi‐type dehydration of precursor **2** using POCl_3_ yielded the isocyanide **3** in 96% yield. With isocyanide **3** in hand, oxazole **4** was obtained via a nonprototropic chain–ring tautomerization under mild acidic conditions [0.01‐M HCl], according to a literature‐reported transformation [18]. This process afforded the desired diene with an overall yield of 72% across the four‐step sequence (Scheme 1B).

**FIGURE 1:**
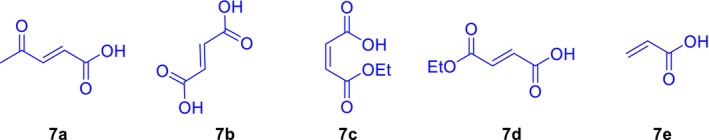
Dienophiles of type **7** used in the inverse‐electron‐demand Diels–Alder approach.

With diene **4** in hand, we turned our attention to the synthesis of the dienophiles: *E*‐4‐oxopentenoic acid (**7a**) [19], fumaric acid (**7b**) [20], and monoethyl maleate (**7c**) [21], following literature‐reported procedures (Scheme S1). Additionally, commercially available dienophiles such as *E*‐4‐ethoxy‐4‐oxobut‐2‐enoic acid (**7d**) and acrylic acid (**7e**) were also employed in subsequent IEDDA cycloaddition reactions (Figure 1).

After synthesizing diene **4** and dienophiles **7a**, **7b**, and **7c**, the corresponding IEDDA reactions were performed. As a model reaction, the cycloaddition between diene **4** and keto acid **7a** to afford compounds **10a** and **10′a** was selected to optimize the reaction conditions (Table 1).

**TABLE 1:** Optimization of the Diels–Alder reaction of diene **4** with keto acid **7a** to afford the compounds **10a** and **10′a**.

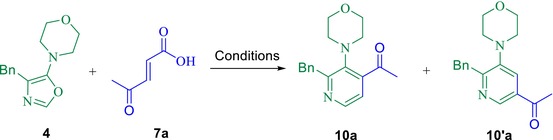				
Entry	Solvent	Reaction time (days)	**10a**:**10′a** ratio	Yield (%)^a^
1	Et_2_O	5	5:2	39
2	THF	3	—	—
3	MeCN	2	—	—
4	CHCl_3_	15	3:5	46

^a^ Yields calculated from the inseparable mixture of regioisomers.

Initial experiments using diethyl ether (Et_2_O) and chloroform (CHCl_3_) as solvents led to the formation of the desired **10a**/**10′a**‐type pyridines in yields of 39% and 46%, respectively. Interestingly, regioselectivity was found to be solvent‐dependent: In Et_2_O, the *para* isomer predominated over the *meta* isomer in a 5:2 ratio (Table 1, Entry 1), whereas in CHCl_3_, the selectivity reversed, favoring the meta isomer with a 3:5 ratio and a slightly higher isolated yield (46%) (Table 1, Entry 4). Notably, the two productive solvents (Et_2_O, ε = 4.3; CHCl_3_, ε = 4.8) possess relatively low dielectric constants, which favor the formation of the N–H–O hydrogen‐bonded complex between diene **4** and the carboxylic acid of the dienophile. In contrast, THF (ε ≈ 7.5) and MeCN (ε ≈ 37.5) are significantly more polar. Their higher dielectric constants enable stronger solvation of the carboxylic acid, which disrupts acid‐base association with oxazole **4**. This prevents formation of the activated protonated/H‐bonded intermediate required for the IEDDA cycloaddition, consistent with the complete absence of product in these solvents. Based on these results, CHCl_3_ was selected as the optimal solvent for further transformations.

**SCHEME 2:**
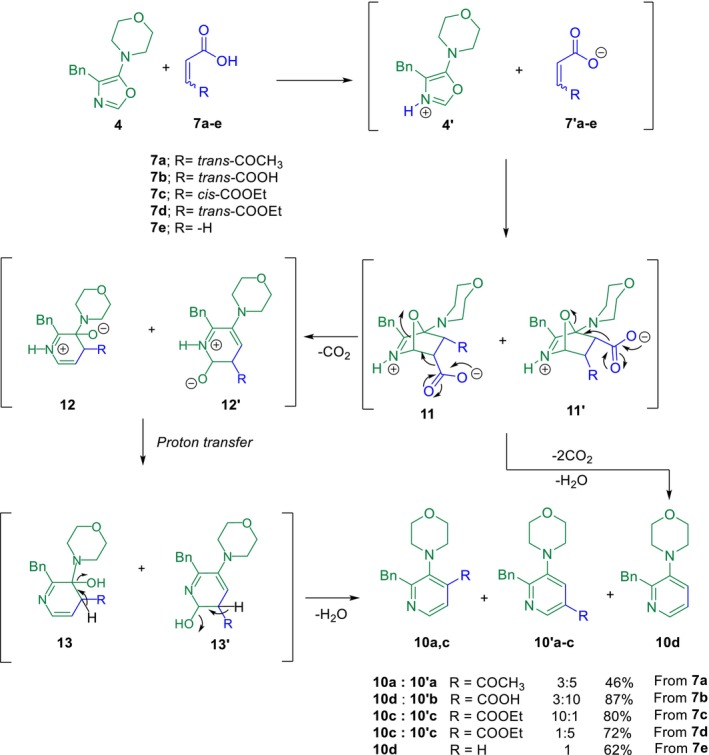
IEDDA cycloadditions between diene **4** and dienophiles **7a**–**e**, performed in CHCl_3_ or CHCl_3_/DMSO (one drop) at room temperature. Full synthetic details are provided in the Supporting Information. The proposed mechanism for the formation of pyridines of type **10** is also depicted.

With the optimized conditions established, we proceeded to carry out the IEDDA cycloadditions between oxazole **4** and dienophiles **7b**–**7e**. In all cases involving dienophiles **7a**, **7c**, and **7d**, the ^1^H NMR analysis of the crude reaction mixtures revealed the formation of regioisomeric mixtures of *para*‐ and *meta*‐substituted pyridine derivatives. These results are consistent with the generation of two regioisomeric intermediates (**11** and **11′**), which undergo sequential decarboxylation and dehydration steps to furnish the aromatic products **10** and **10′**, as illustrated in Scheme 2. In the cycloaddition of oxazole **4** with fumaric acid **7b**, using CHCl_3_ as solvent with the addition of a small drop of DMSO to aid product solubilization, ^1^H NMR analysis indicated a product mixture consisting of *meta*‐substituted pyridine **10′b** and 2‐benzyl‐3‐morpholinylpyridine **10d** in a 10:3 ratio. Full NMR spectra for all compounds are provided in Figures S1–S13. This outcome supports a double decarboxylation pathway for fumaric acid, ultimately leading to the formation of the product **10d**. To further investigate this solvent‐dependent regioselectivity and rationalize the predominance of **10′c** over **10c** from **7d** (*trans* configuration), total electronic energies (including zero‐point corrections) as well as the HOMO–LUMO energy gaps of both regioisomers were calculated in CHCl_3_ using the polarizable continuum model (PCM) at the M06‐2X/6‐311G(d,p) level of theory implemented in GAUSSIAN09 [22]. The results show that **10′c** is thermodynamically more stable than **10c**, fully consistent with the experimentally observed regioisomeric ratio. Full computational procedures and the corresponding energy values are provided in Table S2. Notably, when the compound **7c** (*cis* configuration) was reacted with diene **4**, the reaction proceeded markedly faster than with compound **7d** (*trans* configuration). Upon addition of dienophile **7c**, an immediate color change, evolution of gas (CO_2_), and condensation of water on the inner walls of the reaction vessel were observed, indicating rapid aromatization. This enhanced reactivity is likely due to the reduced steric hindrance of the *cis*‐substituted dienophile **7c**, which facilitates cycloaddition more efficiently than the *trans*‐configured **7d** (see Scheme 2). We propose that the mechanism involves the in situ protonation of diene **4** due to the presence of the carboxylic acid group in dienophiles of type **7**. Once the nitrogen of the oxazole is protonated, the cycloaddition occurs, yielding the cycloadducts **11** or **11′**. This is followed by a decarboxylation step, promoting the formation of intermediates **12** and **12′**. Subsequently, a proton transfer leads to the formation of the products **13** and **13′**, and finally, the release of a molecule of water results in the formation of the desired pyridines of type **10**, which are the most stable products.

Given the proposed protonation step in the mechanism, a further objective was to assess whether in situ protonation facilitates the IEDDA cycloaddition. To this end, we first evaluated the importance of the (‐COOH) substituent in the dienophiles. New DA reactions were therefore carried out using commercially available dienophiles lacking the carboxylic acid group in their structural backbone: methyl 3‐methoxyacrylate (**7f**), diethyl maleate (**7g**), and diethyl fumarate (**7h**) (Scheme S2).

These cycloadditions were performed in a hermetically sealed tube under a nitrogen atmosphere, varying temperature, solvent, and reaction time. However, no reaction was observed under any of the tested conditions (for further details, see Table S1). Although oxazole **4** was employed under high‐temperature conditions (Table S1, Entries 3 and 6), neither cycloadducts nor pyridinic products were obtained. These preliminary results suggest that the absence of the carboxylic acid proton in the dienophile prevents the reaction from proceeding, indicating that protonation is indeed crucial for the cycloaddition. However, this was only an initial indication supporting the proposed mechanism. To further investigate this aspect, ^1^H, ^13^C, and ^15^N NMR spectroscopy was employed to analyze and confirm the protonation of the heterodiene **4** within the proposed IED aza‐Diels–Alder mechanism.

### 2.2 NMR Analysis

**FIGURE 2:**
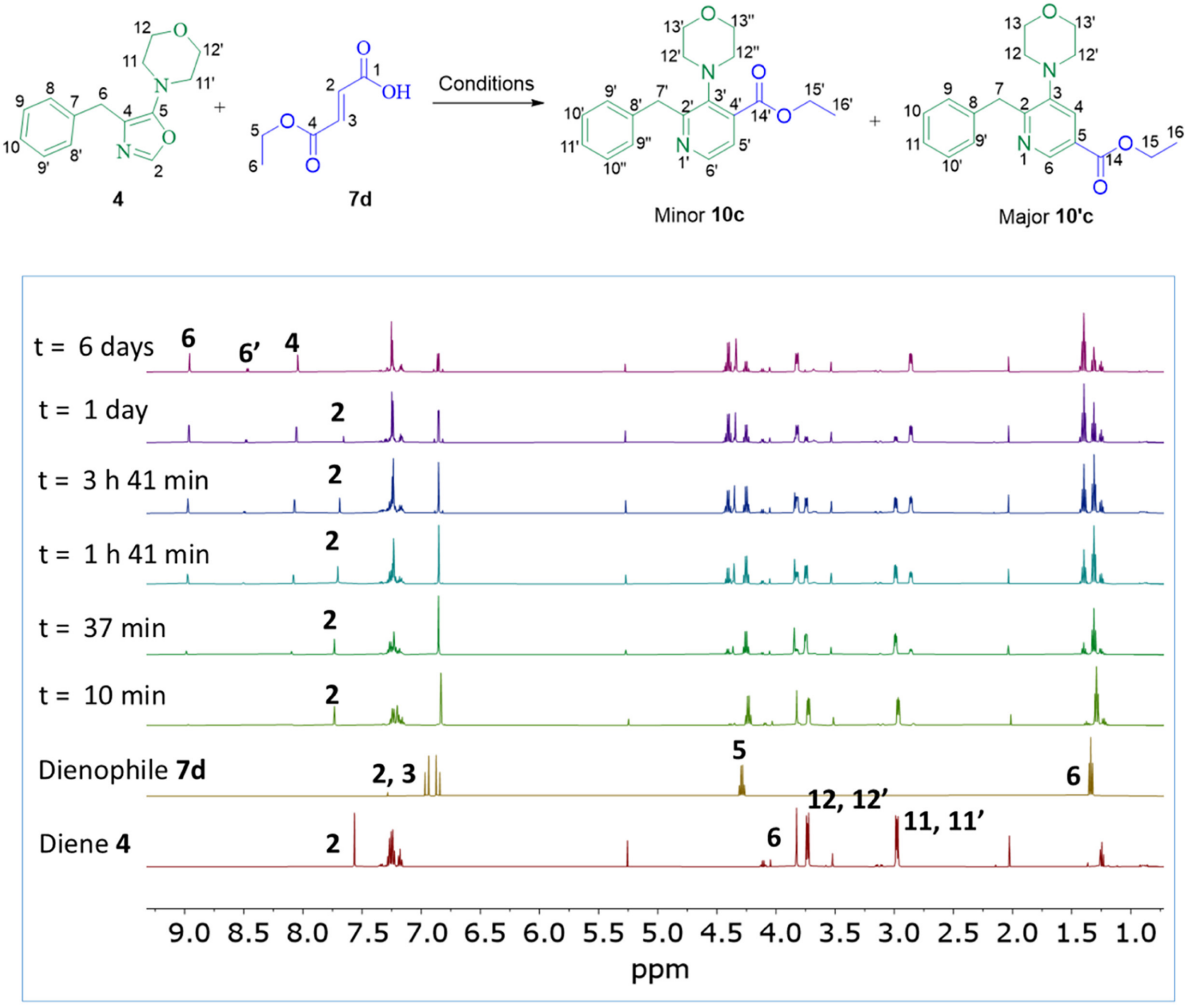
^1^H NMR analysis of product formation in the Diels–Alder reaction of diene **4** and dienophile **7d**, highlighting the temporal evolution of **10′c** and **10c**.

To conduct this study, the DA cycloaddition between the diene **4** and the dienophile **7d** was carried out to analyze the protonation of the nitrogen atom in the diene via NMR spectroscopy. If the heterodiene **4** was protonated by the carboxylic acid group of the dienophile **7d**, we would expect to observe a downfield shift in the carboxylic acid proton (‐COOH, H‐1) as well as the O‐CH‐N proton (H‐2) of the diene **4** in the NMR spectrum. After mixing **4** and **7d** and stirring for 10 min at room temperature, the OH signal of the dienophile **7d** was observed to shift upfield until it underwent chemical exchange with the water produced during the reaction (see Scheme S3), causing overlap with the water signal. This suggests that proton transfer occurs rapidly and cannot be directly observed by the chemical shift of the dienophile. The ^1^H NMR spectrum also revealed signals corresponding to the formation of the pyridine product **10′c** (*meta*) as the major product. After 37 min of reaction, signals associated with the minor product **10c** (*para*) also began to appear (Figure 2). Analysis of the H‐2 (O‐CH‐N) proton in 4 showed a downfield shift, consistent with protonation. Furthermore, the vinylic protons H‐3 (= CH‐COOH) and H‐4 (= CH‐COOEt) were also affected by the presence of diene **4**.

**FIGURE 3:**
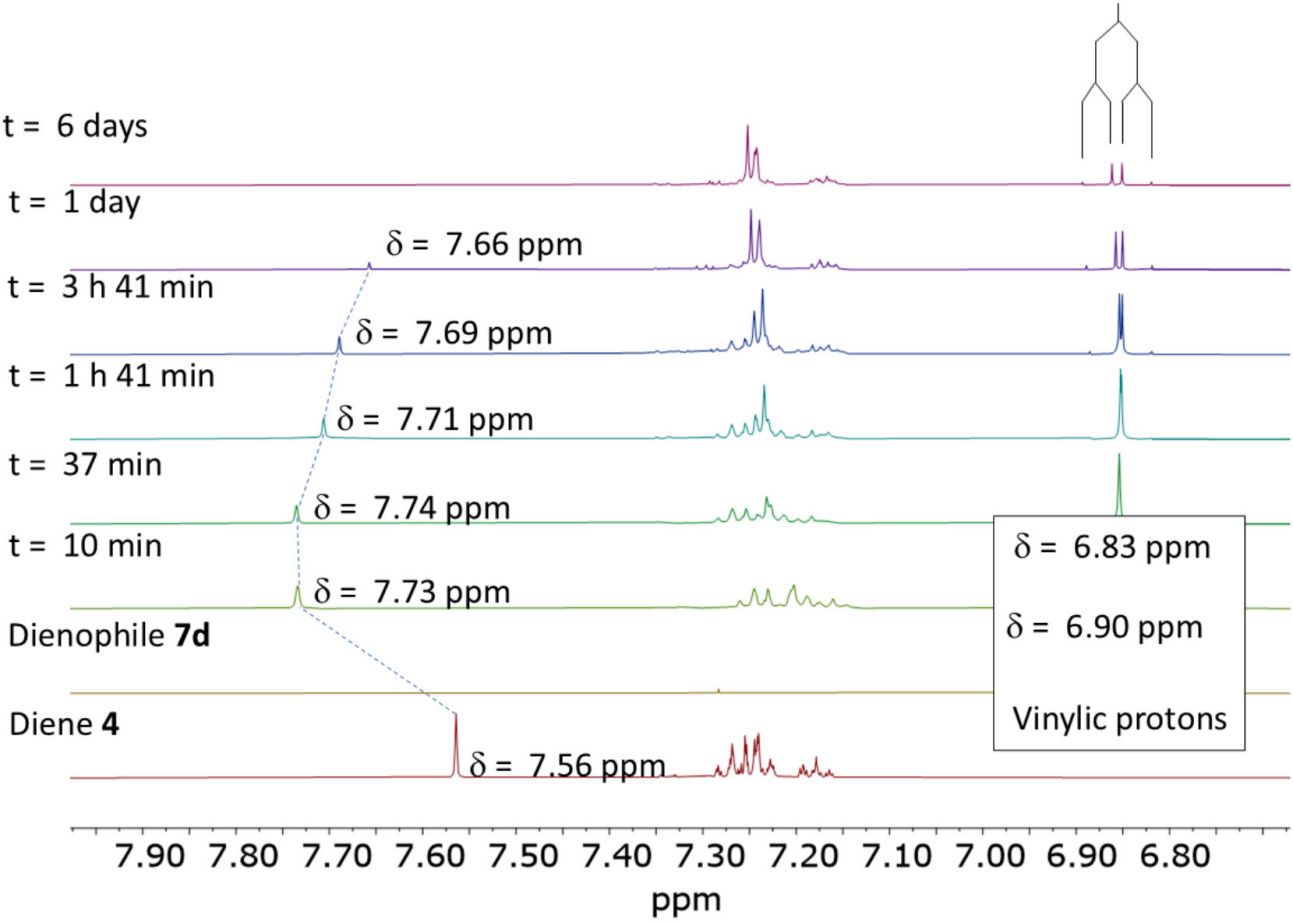
^1^H NMR (500 MHz, CDCl_3_) expansion of the vinylic and aromatic region for the reaction of oxazole **4** with dienophile **7d**.

Figure 3 shows an expanded view of the vinylic and aromatic region of the ^1^H NMR spectrum. Initially, each proton appeared as a doublet (^1^H NMR spectrum of dienophile **7d**, Figure 3); however, they later collapsed into a single signal (^1^H NMR spectrum of t = 10, Figure 3). This behavior suggests that the protons became chemically and magnetically equivalent, likely due to rapid proton exchange between the diene and dienophile prior to the formation of the cycloaddition products. The reaction was continuously monitored by NMR, and after 3 h and 41 min (Figure 3), the vinylic protons regained their differentiation, maintaining this state until the reaction was completed.

**FIGURE 4:**
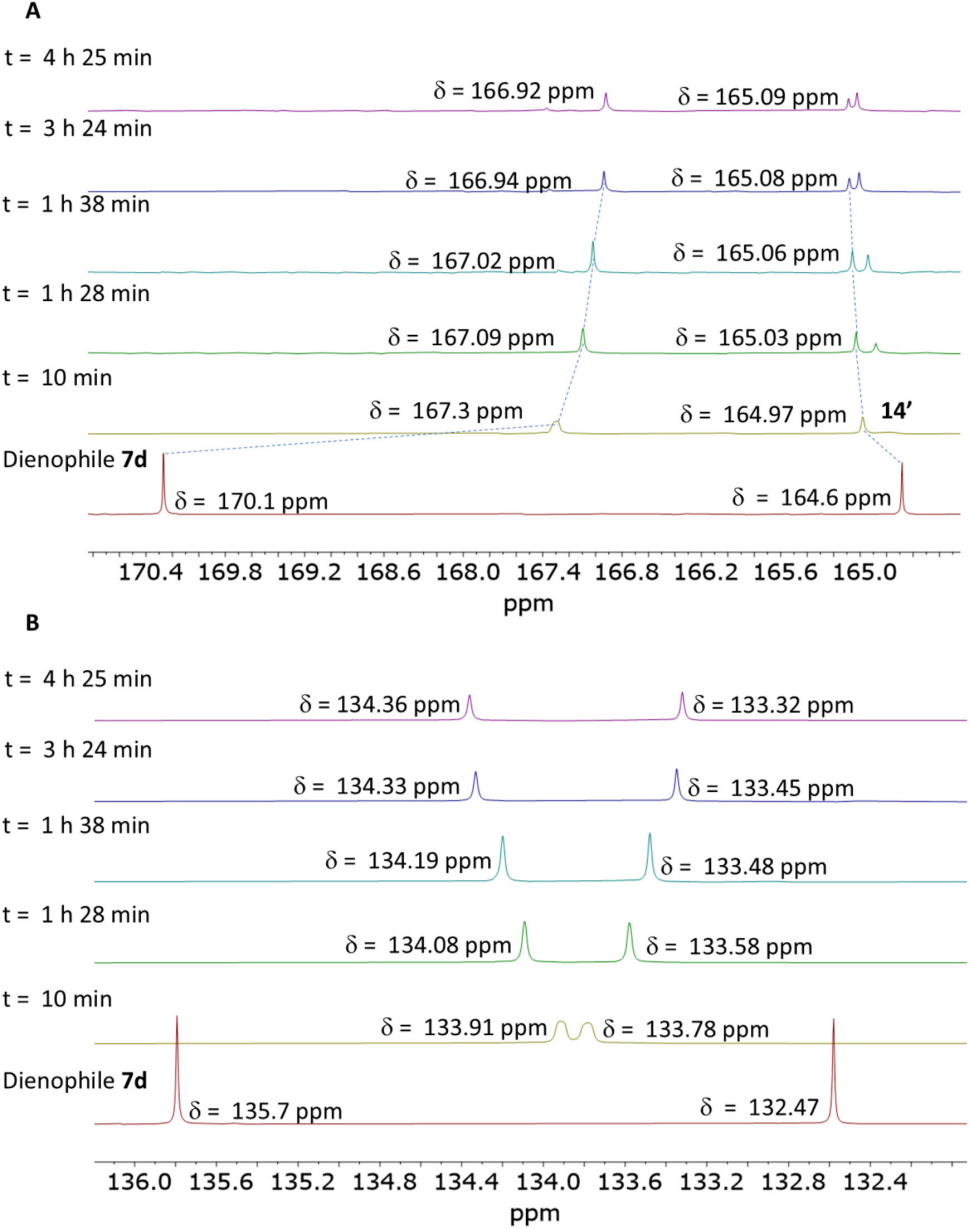
^13^C NMR spectrum (125 MHz, CDCl_3_), magnification of the carbonyl (A) and vinyl (B) carbon signals of **7d** and their corresponding reactions with **4**.

When the ^13^C NMR spectra of the dienophile were analyzed (Figure 4), notable changes were observed in the vinyl and carbonyl carbon signals of dienophile **7d**. The carbonyls are affected first, with C‐2 shifting upfield by approximately 3 ppm (Figure 4A). Then, the vinyl carbons experience shifts, with the C‐3 carbon atom moving upfield by approximately 2 ppm, nearly collapsing, while C‐4 moves downfield by around 1.3 ppm within an approximate reaction time of 10 min (Figure 4B). Nevertheless, after 1 h and 28 min, the vinyl carbon signals differentiate once again (Figure 4B). As previously mentioned, based on our ^1^H NMR chemical shift analysis of both diene **4** and dienophile **7d**, we consider that proton transfer is likely so fast that it is also undetectable by ^13^C NMR. Therefore, a ^15^N NMR study was conducted to gain a deeper understanding of this reaction. Since ^15^N NMR spectroscopy is highly sensitive to the electronic properties of atoms surrounding nitrogen, it has proven useful for elucidating the structures of organic, organometallic, inorganic, and biochemical compounds that are otherwise difficult to characterize [23]. Thus, ^15^N NMR provides valuable information on the chemical environment surrounding the nitrogen atom through its chemical shift, allowing us to determine whether the protonation of nitrogen in diene **4** had occurred.

The study began with the ^15^N NMR experiment of diene **4** (Figure S20 and Table S4) using ammonia as a reference (50.66 MHz, *J* ^*3*^_(1H, 15N)_ = 3 Hz, CDCl_3_). The ^15^N NMR spectrum shows two distinct ^15^N signals, corresponding to the two nitrogen atoms in the diene.

The nitrogen atom in the oxazole moiety (**4**) exhibits a chemical shift of δ = 262.48 ppm, which falls within the range reported in the literature for nitrogen atoms in azoles (δ^15^N = 230–330 ppm) [23]. Meanwhile, the nitrogen in the morpholine displayed a shift of δ = 39.47 ppm, consistent with previously reported values for alkylamines (δ^15^N = 0–70 ppm) [22]. Once the chemical shifts were determined, the dienophile **7d** was added in a 1:1 ratio, and the ^15^N NMR kinetic study was conducted. The chemical shifts corresponding to the two nitrogen atoms in the diene are presented in Table 2.

**TABLE 2:** ^15^N NMR chemical shifts (δ, ppm) at 50.66 MHz, ^*3*^ *J*(^1^H,^15^N) = 3 Hz, CDCl_3_.

δ^15^N	t = 0	t = 47 min	t = 1 h 28 min	t = 2 h 14 min
*N*‐morpholine	39.47	38.32	38.75	38.75
*N*‐oxazole	262.48	255.89	256.38	257.57

Comparing the ^15^N NMR chemical shifts of heterodiene **4** before and after the addition of dienophile **7d**, we observed that *N*‐oxazole atom in **4** shifted downfield by approximately δ^15^N ≈ 7 ppm, while the *N*‐morpholine only shifted by δ^15^N ≈ 1 ppm. After 1 h and 28 min, an increase in the concentration of product **10′c** was detected, allowing us to observe two new ^15^N NMR signals corresponding to the nitrogen atoms in morpholine and in the major pyridine‐derived product **10′c**. The chemical shifts are summarized in Table 3.

**TABLE 3:** ^15^N NMR chemical shifts (δ, ppm) at 50.66 MHz, ^*3*^ *J*(^1^H,^15^N) = 15 Hz, CDCl_3_ for **10′c**.

δ^15^N	t = 1 h 28 min	t = 2 h 14 min	t = 21 h	t = 7 days
*N*‐morpholine	48.38	48.99	49.00	49.14
*N*‐pyridine	302.51	304.39	309.29	311.43

At the end of the reaction, we noted that the ^15^N chemical shift of the nitrogen atom in morpholine moiety in **4** had shifted downfield by Δδ = 10 ppm, while the nitrogen in pyridine within **10′c** exhibited a final shift of δ = 311.43 ppm, consistent with literature values for pyridine nitrogen atoms (δ^15^N = 230–330 ppm) [22]. However, after analyzing these results, new questions came regarding the nature of chemical shift changes observed during the reaction study. Could these shifts be attributed to the protonation of diene **4** by the acidic proton of dienophile **7d** (–COOH, H‐1)? Alternatively, could they result from a hydrogen‐bonding interaction (N···H···O), or are they simply a consequence of π–π stacking interactions? To attend them, the ^1^H and ^13^C NMR analysis of dienophile **7d** at different concentrations allowed us to rule out that the observed chemical shift changes were due to protonation of **4**, rather than concentration effects (see Table S3 and Figures S14–S19 for further details). It was observed that the H‐1 (‐COOH) proton shifted upfield to lower concentrations, suggesting the presence of intermolecular hydrogen bonding [24]. The vinyl protons H‐3 and H‐4 showed no significant changes. Likewise, the ^13^C NMR spectra did not reveal any variations in chemical shifts. Thus, we rule out that the changes in the chemical shifts are a result of concentration in the reaction between **4** and **7d**. Subsequently, we evaluated whether the interaction between diene **4** and dienophile **7d** corresponded to protonation, an N–H–O hydrogen bond, or π–π stacking interactions. The ^1^H NMR study revealed a downfield shift for the H‐1 (‐COOH) proton of the dienophile and the H‐2 (O‐CH‐N) proton of the diene, suggesting either a protonation event or hydrogen‐bonding interaction. In the ^13^C NMR spectrum, C‐2 of the carboxyl group shifted upfield, while C‐5 shifted downfield, indicating an interaction with the diene. The ^15^N NMR spectrum showed shifts consistent with protonated species; however, the lack of reaction after 7 days suggests that an N–H–O hydrogen bond is present rather than effective protonation. To differentiate between π–π stacking and hydrogen bonding, additional studies were conducted using benzene, morpholine, and pyridine in the presence of dienophile **7d**. The results indicate that hydrogen bonding occurs with morpholine, whereas pyridine undergoes immediate protonation. When nitrogen was removed from the system, the vinyl protons of the dienophile showed no changes, ruling out π–π stacking as a key factor in the reaction. In conclusion, it was determined that the N–H–O hydrogen bond interaction between **4** and dienophile **7d** plays a crucial role in facilitating the IEDDA cycloaddition. Finally, to distinguish whether the reaction proceeds via hydrogen bonding or through heterocycle protonation, we carried out a titration experiment of oxazole **4a** with trifluoroacetic acid (TFA), followed by ^1^H and ^15^N NMR analysis.

### 2.3 Oxazole (4a) Titrations

To understand the trends observed in the ^15^N chemical shifts in the previous reactions, a gradual titration of oxazole **4a** with TFA was performed. The ^1^H NMR results are presented in Table 4.

**TABLE 4:** ^1^H NMR chemical shifts (δ, ppm) (500 MHz, D_2_O).

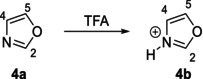				
δ^1^H	36 μL^a^	14‐μL TFA	28‐μL TFA	42‐μL TFA
H‐2	8.20	8.68	9.42	9.67
H‐4	7.22	7.43	7.75	7.88
H‐5	7.92	8.07	8.32	8.41

^a^ Initial oxazole **4a** concentration.

In general, the proton signals of oxazole **4a** shifted progressively with the addition of TFA. However, the most notable shift was observed for proton H‐2, which moved downfield from 8.20 to 9.67 ppm (Δδ ≈ 1.5 ppm), likely due to the positive charge developed on the nitrogen atom upon protonation of the heterocycle. Notably, no further changes in chemical shift were observed even with additional TFA. Similarly, ^15^N NMR spectra were analyzed and compared for the neutral oxazole **4a** and its protonated oxazolium cation **4b**, revealing noteworthy results (Table 5).

**TABLE 5:** ^15^N NMR chemical shifts (δ, ppm) (50.66 MHz, D_2_O).

Compound	Initial	14‐μL TFA	28‐μL TFA	42‐μL TFA
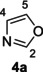	246.05	228.30	201.44	192.40 (193.40)^a^

^a^ Chemical shift of compound **4b**.

It was remarkable to observe that the final chemical shift obtained at the end of the titration corresponded to the ^15^N signal of the oxazolium cation **4b**. Moreover, as seen in the previous reactions, the same trend in ^15^N shifts was observed: initially, a high‐frequency chemical shift, which progressively moved upfield (lower frequency) due to the presence of an acidic proton.

## 3 Conclusions

In closing, we successfully developed an IEDDA Kondrat'eva aza‐Diels–Alder cycloaddition for the synthesis of highly substituted pyridines using an oxazole‐derived diene obtained via an Ugi–Zhu multicomponent reaction. The regiochemical and stereochemical outcomes of the cycloadditions were found to be influenced by the nature of the dienophiles and the reaction conditions. The presence of a carboxyl group in the dienophile proved to be essential for the reaction, as control experiments with noncarboxylated dienophiles failed to yield the expected products. Kinetic studies using NMR spectroscopy provided crucial insights into the reaction mechanism, particularly the role of in situ protonation in facilitating the cycloaddition. The observed shifts in ^1^H, ^13^C, and ^15^N NMR spectra confirmed that protonation of the oxazole nitrogen played a key role in promoting the IEDDA process. Additionally, the reaction kinetics were affected by the electronic properties of the dienophile, with *cis*‐configured dienophiles reacting more rapidly than their *trans* counterparts. Overall, our findings highlight the efficiency of the IEDDA reaction for constructing complex pyridine frameworks and underscore the importance of N–H–O hydrogen bond and subsequent nitrogen protonation in the reaction mechanism. These results contribute to a deeper understanding of aza‐Diels–Alder chemistry and expand the synthetic toolkit for the preparation of functionalized pyridine derivatives with potential applications in medicinal chemistry.

## Conflicts of Interest

The authors declare no conflicts to interest.

## Supporting information




**Scheme S1.** Synthetic approaches for the synthesis of dienophiles **7a**, **7b**, and **7c** (A–C), and the commercially available dienophiles **7d** and **7e** (D).
**Scheme S2.** IEDDA cycloadditions without protonated dienophiles.
**Table S1:** Diels–Alder cycloadditions of diene **4** with different dienophiles **7f–h** lacking the carboxylic acid group.
**Table S2:** Calculated HOMO–LUMO gaps and total electronic energies for **10c** and **10′c** in CHCl_3_ (using the polarizable continuum model (PCM) with zero‐point energy correction) at the M06‐2X/6‐311G(d,p) level of theory.
**Figure S1:** mrc70075‐sup‐0001‐Supporting_Information.docx. ^1^H NMR spectrum of 2‐Isocyano‐1‐morpholino‐3‐phenylpropan‐1‐one (**3**) in CDCl_3_ at 500 MHz.
**Figure S2:** mrc70075‐sup‐0001‐Supporting_Information.docx. ^13^C NMR spectrum of 2‐Isocyano‐1‐morpholino‐3‐phenylpropan‐1‐one (**3**) in CDCl_3_ at 126 MHz.
**Figure S3:** mrc70075‐sup‐0001‐Supporting_Information.docx. ^1^H NMR spectrum of 2‐Isocyano‐1‐morpholino‐3‐phenylpropan‐1‐one (**3**) in CDCl_3_ at 500 MHz.
**Figure S4:** mrc70075‐sup‐0001‐Supporting_Information.docx. ^1^H NMR spectrum (500 MHz, CDCl_3_) of the diastereomeric mixture of **10a** and **10′a** that only shows the ^1^H signals of **10a**.
**Figure S5:** mrc70075‐sup‐0001‐Supporting_Information.docx. ^13^C NMR spectrum (125 MHz, CDCl_3_) of the diastereomeric mixture of **10a** and **10′a** that only shows the ^13^C signals of **10a**.
**Figure S6:** mrc70075‐sup‐0001‐Supporting_Information.docx. ^1^H NMR spectrum (500 MHz, CDCl_3_) of the diastereomeric mixture of **10a** and **10′a** that only shows the ^1^H signals of **10′a**.
**Figure S7:** mrc70075‐sup‐0001‐Supporting_Information.docx. ^13^C NMR spectrum (125 MHz, CDCl_3_) of the diastereomeric mixture of **10a** and **10′a** that only shows the ^13^C signals of **10′a**.
**Figure S8:** mrc70075‐sup‐0001‐Supporting_Information.docx. ^1^H NMR spectrum (500 MHz, CDCl_3_) of the diastereomeric mixture of **10c** and **10′c** that only shows the ^1^H signals of **10′c**.
**Figure S9:** mrc70075‐sup‐0001‐Supporting_Information.docx. ^13^C NMR spectrum (125 MHz, CDCl_3_) of the diastereomeric mixture of **10c** and **10′c** that only shows the ^13^C signals of **10′c**.
**Figure S10:** mrc70075‐sup‐0001‐Supporting_Information.docx. ^1^H NMR spectrum (500 MHz, CDCl_3_) of **10d**.
**Figure S11:** mrc70075‐sup‐0001‐Supporting_Information.docx. ^13^C NMR spectrum (125 MHz, CDCl_3_) of **10d**.
**Figure S12:** mrc70075‐sup‐0001‐Supporting_Information.docx. ^1^H NMR spectrum (500 MHz, CDCl_3_) of **10′b**.
**Figure S13:** mrc70075‐sup‐0001‐Supporting_Information.docx. ^13^C NMR spectrum (125 MHz, CDCl_3_) of **10′b**.
**Scheme S3.** Expanded region of the ^1^H NMR spectrum (500 MHz, CDCl_3_) illustrating the kinetics of the Diels–Alder cycloaddition between diene **4** and dienophile **7d**. Only the 9–12 ppm region is shown to highlight the proton transfer from **7d** to **4**.
**Table S3:** Chemical shifts (δ, ppm) from ^1^H NMR (500 MHz, CDCl_3_) of compound **7d** at different concentrations.
**Figure S14:** Intermolecular hydrogen bonding in compound **7d**.
**Figure S15:** mrc70075‐sup‐0001‐Supporting_Information.docx. ^1^H NMR spectrum (500 MHz, CDCl_3_) of acid **7d**.
**Figure S16:** mrc70075‐sup‐0001‐Supporting_Information.docx. ^13^C NMR spectrum (125 MHz, CDCl_3_) of acid **7d**.
**Figure S17:** mrc70075‐sup‐0001‐Supporting_Information.docx. ^1^H NMR spectrum (500 MHz, CDCl_3_) of the cycloaddition reaction between diene **4a** and dienophile **7d**. (A) Expanded region from 10 to 12 ppm highlighting the chemical shift of the acidic proton H‐1. (B) Chemical structures of the diene and dienophile and (C) cycloaddition reaction between **4a** and **7d**, with ^1^H NMR spectra recorded at T = 13 h (blue) and T = 13.41 h (purple).
**Figure S18:** Magnification of the ^1^H NMR spectrum (500 MHz, CDCl_3_) showing chemical shift changes in ppm.
**Figure S19:** Magnification of ^13^C NMR spectrum (125 MHz, CDCl_3_) showing chemical shift changes in ppm.
**Figure S20:** mrc70075‐sup‐0001‐Supporting_Information.docx. ^15^N NMR spectrum (50.66 MHz, *J* ^*3*^_1H,15N_ = 3 Hz, CDCl_3_) of oxazole **4**, using ammonia as a reference.
**Table S4:** mrc70075‐sup‐0001‐Supporting_Information.docx. ^15^N NMR chemical shifts (δ, ppm; 50.66 MHz, CDCl_3_).

## Data Availability

The data supporting this article have been included in the Supporting Information.
