# Supplementary material for: NMR‐Based Structural Analysis of Highly Substituted Pyridines From Kondrat'eva Aza‐Diels–Alder Cycloadditions
Source: Magn Reson Chem. 2025 Dec 18;64(3):324–32. doi: 10.1002/mrc.70075 (PMC12867586; doi:10.1002/mrc.70075)
Supplement: Supplementary file 1 — Scheme S1. Synthetic approaches for the synthesis of dienophiles 7a, 7b, and 7c (A–C), and the commercially available dienophiles 7d and 7e (D). Scheme S2. IEDDA cycloadditions without protonated dienophiles. Table S1: Diels–Alder cycloadditions of diene 4 with different dienophiles 7f–h lacking the carboxylic acid group. Table S2: Calculated HOMO–LUMO gaps and total electronic energies for 10c and 10′c in CHCl3 (using the polarizable continuum model (PCM) with zero‐point energy correction) at the M06‐2X/6‐311G(d,p) level of theory. Figure S1: mrc70075‐sup‐0001‐Supporting_Information.docx. 1H NMR spectrum of 2‐Isocyano‐1‐morpholino‐3‐phenylpropan‐1‐one (3) in CDCl3 at 500 MHz. Figure S2: mrc70075‐sup‐0001‐Supporting_Information.docx. 13C NMR spectrum of 2‐Isocyano‐1‐morpholino‐3‐phenylpropan‐1‐one (3) in CDCl3 at 126 MHz. Figure S3: mrc70075‐sup‐0001‐Supporting_Information.docx. 1H NMR spectrum of 2‐Isocyano‐1‐morpholino‐3‐phenylpropan‐1‐one (3) in CDCl3 at 500 MHz. Figure S4: mrc70075‐sup‐0001‐Supporting_Information.docx. 1H NMR spectrum (500 MHz, CDCl3) of the diastereomeric mixture of 10a and 10′a that only shows the 1H signals of 10a. Figure S5: mrc70075‐sup‐0001‐Supporting_Information.docx. 13C NMR spectrum (125 MHz, CDCl3) of the diastereomeric mixture of 10a and 10′a that only shows the 13C signals of 10a. Figure S6: mrc70075‐sup‐0001‐Supporting_Information.docx. 1H NMR spectrum (500 MHz, CDCl3) of the diastereomeric mixture of 10a and 10′a that only shows the 1H signals of 10′a. Figure S7: mrc70075‐sup‐0001‐Supporting_Information.docx. 13C NMR spectrum (125 MHz, CDCl3) of the diastereomeric mixture of 10a and 10′a that only shows the 13C signals of 10′a. Figure S8: mrc70075‐sup‐0001‐Supporting_Information.docx. 1H NMR spectrum (500 MHz, CDCl3) of the diastereomeric mixture of 10c and 10′c that only shows the 1H signals of 10′c. Figure S9: mrc70075‐sup‐0001‐Supporting_Information.docx. 13C NMR spectrum (125 MHz, CDCl3) of the diastereomeric mixture of 10c and [file MRC-64-324-s001.docx]

***Supporting Information***

**NMR-Based Structural Analysis of Highly Substituted Pyridines from Kondrat’eva Aza-Diels–Alder Cycloadditions**

Galdina V. Suárez-Moreno,^a^ Francisco Méndez,^b*^ Atilano Gutierrez-Carrillo,^b^ Mónica A. Rincón-Guevara,^c^ Yoarhy A. Amador-Sánchez,^b*^ Alejandro Islas-Jácome,^b*^ and Eduardo González-Zamora^b*^

*^a^* *Instituto Politécnico Nacional, Unidad Profesional Interdisciplinaria de Biotecnología, Av. Acueducto S/N, C.P. 07340 Ciudad de México, México.*

*^b^ Departamento de Química, Universidad Autónoma Metropolitana-Iztapalapa, Av. Ferrocarril San Rafael Atlixco 186, Col. Leyes de Reforma 1A Sección, Iztapalapa, 09310, Ciudad de México, México.*

*^c^ Laboratorio Divisional de Espectrometría de Masas, División de Ciencias Biológicas y de la Salud, Universidad Autónoma Metropolitana, Unidad Iztapalapa, Iztapalapa, CDMX, México.*

| General remarks | S2 |
| --- | --- |
| Synthesis of Diene **4** (4-Benzyl-5-(4-morpholinyl)oxazole) | S3-S4 |
| Synthesis of Dienophiles of type **7** | S5-S6 |
| Synthesis of Diels-Alder adducts | S6-S8 |
| Protonation Studies of Diene **4** in IEDDA Cycloadditions | S9 |
| Theoretical calculations | S10-S12 |
| ^1^H and ^13^C NMR of all synthetized compounds | S13-S25 |
| ^1^H NMR Analysis of Proton Transfer During the Cycloaddition | S26 |
| NMR experiments at different dienophile concentrations | S27-S29 |
| Protonation or N–H···O Hydrogen Bonding Interaction vs π–π Stacking Interaction | S30-S32 |
| ^15^N NMR Spectroscopy | S33-S34 |
| References | S35 |

**General remarks**

All reagents and solvents were obtained from Sigma-Aldrich-Merck and used as received, without further purification, distillation, or drying. **^1^H**, **^15^N**, and **^13^C** Nuclear Magnetic Resonance (NMR) spectra were acquired on a Bruker AMX Advance III spectrometer (500 MHz, Fällande, Uster, Switzerland) using chloroform (CDCl_3_) as solvent. Chemical shifts (δ) are reported in parts per million (ppm) relative to Si(CH_3_)_4_. Coupling constants (*J*) are reported in hertz (Hz). Peak multiplicity is indicated as follows: s = singlet, d = doublet, t = triplet, m = multiplet, bs = broad signal for proton spectra. The spectra were processed using MestReNova software, version 12.0.0-20080 (A Coruña, Spain). **FT-IR spectra** were recorded on a Perkin-Elmer GX FT-IR spectrometer, and the samples were analyzed in potassium bromide (KBr) pellets. The data were expressed in wavenumbers ῡ (cm⁻¹). **High-Resolution Mass Spectrometry** (HRMS) data were acquired using a JEOL MStation JMS-700 and a JEOL Gcmate II. **Elemental analysis** (CHNS/O) was carried out on a "Series II 2400" elemental analyzer. **The reaction progress** was monitored by thin-layer chromatography (TLC) on aluminum sheets coated with silica gel, which were observed under a UV lamp (Cole-Parmer model 9825, 6 watts). **The purification of the final products** was carried out by flash column chromatography using Merck 60 silica gel (230–400 mesh) and by preparative thin-layer chromatography (TLC) on 20 × 20 cm glass plates coated with MN G/UV silica gel 60 containing a UV indicator (F254), employing hexane/ethyl acetate as the mobile phase. The collected fractions were monitored by TLC, with spots visualized under ultraviolet (UV) light at 254 or 365 nm. **Chemical structures** were generated using ChemDraw software, version 15.0.0.106 Professional (Perkin Elmer Informatics, Cambridge, MA, USA).

**Organic Synthesis**

Synthesis of Diene **4** (4-Benzyl-5-(4-morpholinyl)oxazole)

*N*-Formyl phenylalanine.
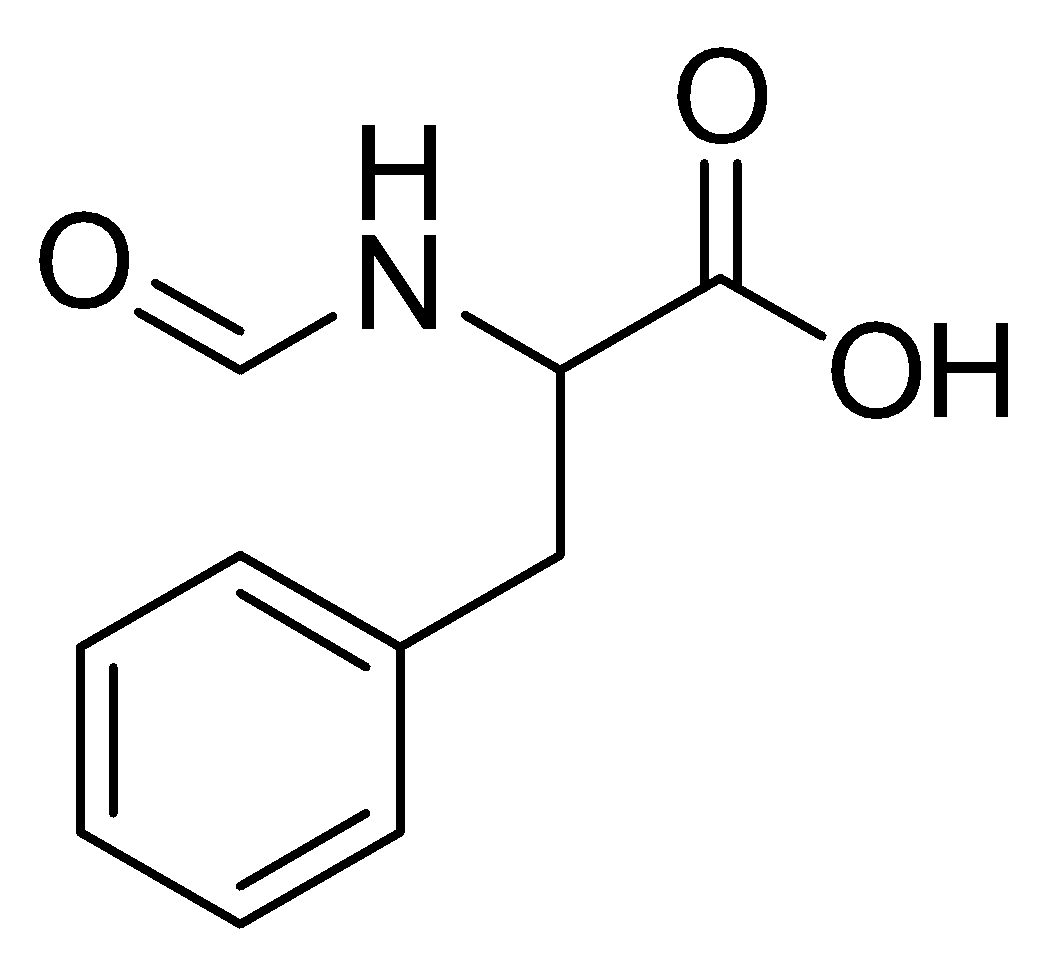


In a 250 mL round-bottom flask, 4.13 g of phenylalanine (25.0 mmol, 1 equiv.) were suspended in 50 mL of formic acid at 0 °C. Subsequently, 17 mL of Ac_2_O (180.0 mmol, 7.2 equiv.) were added dropwise to the mixture under stirring. The reaction mixture was stirred for 2 hours at room temperature. After this time, 20 mL of a water/ice mixture were added, and the reaction mixture was concentrated until dryness. The crude reaction product was used for the next step without purification. A total of 4.65 g (96%) of a grayish-white powder were obtained.

*N*-(1-Morpholino-1-oxo-3-phenylpropan-2-yl)formamide (**2**)
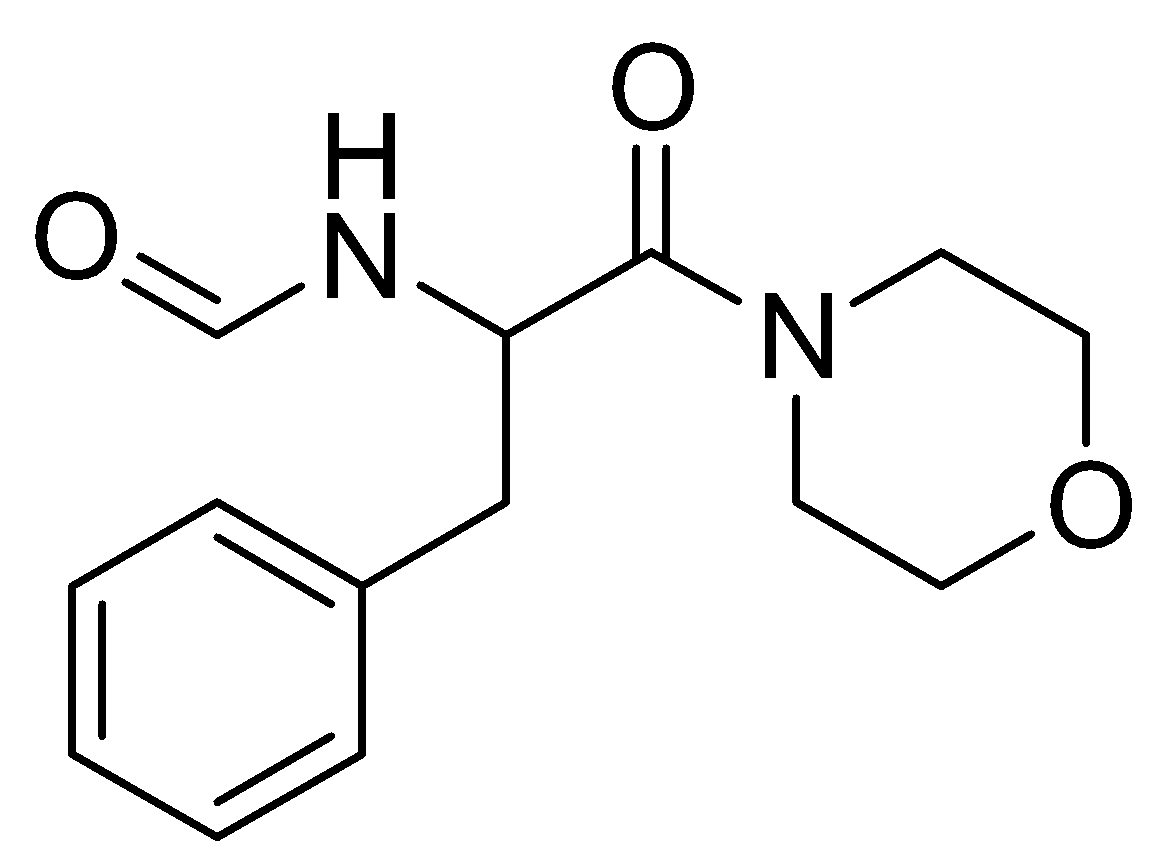


In a 250 mL round-bottom flask, 4.5 g (23.3 mmol, 1.0 equiv.) of the product obtained in the previous step were diluted in 80 mL of dry THF. The mixture was cooled to –25 °C under an inert gas atmosphere, and 2.5 mL of ethyl chloroformate (25.6 mmol, 1.1 equiv.) and 4.3 mL of Et_3_N (30.3 mmol, 1.3 equiv.) were added. After 30 minutes, 2.5 mL of morpholine (27.9 mmol, 1.2 equiv.) were added, and the reaction mixture was stirred at –25 °C for one hour, then at room temperature for 16 hours. After this time, a saturated NaHCO_3_ solution was added, and the mixture was extracted with 3 × 50 mL of AcOEt. The organic phase was washed with a saturated NaCl solution, dried over anhydrous Na_2_SO_4_, filtered using a porous membrane-glass funnel, concentrated to dryness, and the crude reaction product was used in the next step without purification. A total of 5.29 g (89%) of a yellow oil were obtained.

2-Isocyano-1-morpholino-3-phenylpropan-1-one (**3**)

In a 250 mL round-bottom flask, 4.8 g of the product obtained in the previous step were diluted in 90 mL of DCM under an inert gas atmosphere with constant stirring. The temperature was lowered to –25 °C, 15.5 mL of Et_3_N (111.2 mmol, 6.1 equiv.) were added, and the mixture was stirred for 10 minutes. Subsequently, 2.7 mL of POCl_3_ (28.9 mmol, 1.6 equiv.) were added dropwise, and the reaction mixture was stirred for 3 hours at –25 °C. After this time, a saturated K_2_CO_3_ aqueous solution was added, and the mixture was extracted with 3 × 50 mL of DCM. The organic phase was washed with a saturated NaCl aqueous solution, dried over anhydrous Na_2_SO_4_, filtered using a porous membrane-glass funnel, concentrated to dryness, and purified by flash-column chromatography using an AcOEt:Hex 2:3 (v/v) mixture as mobile phase. A total of 3.0 g of a white crystalline solid was obtained with a isolated yield of 61%. *R_f_* = 0.40 (AcOEt:Hex 2:3 v/v); **^1^H NMR (500 MHz, CDCl_3_):** δ 7.37–7.24 (m, 5H, H-8, H-9, H-10, H-11, H-12), 4.54 (dd, ^1^*J* = 6.7 Hz and ^2^*J* = 7.9 Hz, 1H, H-1), 3.71–3.17 (m, 10H, H-6, H-13, H-14, H-16, H-17). **^13^C NMR (126 MHz, CDCl_3_):** δ 163.4 (C-2), 160.1 (C-18), 135.0 (C-7), 129.4 (C-9, C-11), 128.8 (C-8, C-12), 127.7 (C-10), 66.4 (C-1), 46.3 (C-14, C-16), 42.9 (C-13, C-17), 39.0 (C-6).

Synthesis of 4-Benzyl-5-(4-morpholinyl)oxazole (**4**)

In a 50 mL round-bottom flask equipped with a magnetic stirrer, isocyanide **3** (0.5 g, 2.05 mmol) was placed in CH_2_Cl_2_ (8.5 mL), followed by the addition of a 0.05 M HCl solution. The reaction mixture was stirred until a light-yellow solution was observed. Upon completion of the reaction, the mixture was concentrated under vacuum, and the product was purified by column chromatography using silica gel with Hex:AcOEt 4:1 (v/v) as the mobile phase. The product was obtained as a light-yellow oil with a 90% isolated yield. **¹H NMR (500 MHz, CDCl₃):** δ = 7.59 (s, 1H, H-2), 7.20–7.32 (m, 5H, Ph), 3.85 (s, 2H, H-6), 3.75–3.77 (m, 4H, H-12 and H-12’), 2.99–3.01 (m, 4H, H-11 and H-11’). **¹³C NMR (125 MHz, CDCl₃):** δ = 151.9 (C-5), 146.3 (C-2), 139.4 (C-7), 124.5 (C-4), 128.5 (C-8 and C-8’), 128.5 (C-9 and C-9’), 126.3 (C-10), 66.9 (C-12 and C-12’), 51.1 (C-11 and C-11’), 31.8 (C-6).

Synthesis of Dienophiles of type **7**: *E*-4-oxopentenoic acid (**7a**), fumaric acid (**7b**), and monoethyl maleate (**7c**),

Compound **7a** was synthesized in two steps following the protocol reported by Salomón *et al.*, beginning with the oxidation of 2-methylfuran (**5**) to the intermediate butenolide **6**, followed by pyridine-mediated ring opening, yielding the product **7a** in 80% isolated yield (Scheme S1A). Fumaric acid (**7b**) was obtained by acid hydrolysis of its corresponding monoester, affording a 90% yield (Scheme S1B). Meanwhile, monoethyl maleate (**7c**) was synthesized in a single step ethanolysis of commercially available maleic anhydride (**9**) following the procedure reported by Powers *et al.*, with an 80% yield (Scheme S1C).

**Scheme S1**. Synthetic approaches for the synthesis of dienophiles **7a**, **7b** and **7c** (A-C), and the commercially available dienophiles **7d** and **7e** (D).

Synthesis of *E*-4-oxo-pentenoic acid synthesis (acetylacrylic acid, **7a**)

Compound **7a** was prepared according to previously reported literature.^1^ **^1^H NMR (500 MHz, CDCl_3_):** δ = 9.45 (s, 1H, H-1), 7.11 (d, *J* = 16.0 Hz, 1H, H-3), 6.67 (d, *J* = 16.0 Hz, 1H, H-4), 2.41 (s, 3H, H-6).

Synthesis of fumaric acid (**7b**)

Compound **7b** was prepared according to previously reported literature.^3^ Melting point: 278–280°C. **^1^H NMR (500 MHz, CDCl_3_):** δ = 13.0 (s, 1H, OH), 6.78 (d, *³J₃*H,₄H = 15.7 Hz, 1H, H-2), 6.48 (d, *³J₃*H,₄H = 15.7 Hz, 1H, H-3).

Synthesis of monoethyl maleate (**7c**)

Compound **7c** was prepared according to previously reported literature.^3^ **^1^H NMR (500 MHz, CDCl_3_)**: δ = 10.99 (s, 1H, OH), 6.30 (s, 2H, H-2 and H-3), 4.26 (q, *J* = 7.1 Hz, 2H, H-5), 1.3 (t, *J* = 7.1 Hz, 3H, H-6).

**Synthesis of Diels-Alder Adduct**s.

Synthesis of 4-acetyl-2-benzyl-3-morpholinylpyridine (**10a**) and 3-Acetyl-6-benzyl-5-morpholinylpyridine (**10'a**)

In a 50 mL round-bottom flask equipped with a magnetic stirrer, 4-benzyl-5-(4-morpholinyl)oxazole **4** (0.1 g, 0.41 mmol) was dissolved in CHCl_3_ (4 mL). After that, the dienophile **7a** (0.05 g, 0.41 mmol) was added. The solution was stirred for 15 days at room temperature. After the reaction was complete, the solvent was evaporated. The crude product was purified by column chromatography using silica gel with Hex:AcOEt 5:1 (v/v) as the eluent, yielding the compounds **10a** and **10’a** as yellow oil. NMR analysis revealed an inseparable mixture of regioisomers para and meta **10a** and **10'a**, respectively in a 3:5 ratio, with a 46% yield of the purified mixture.

**^1^H NMR (500 MHz, CDCl_3_) 10a**: δ = 8.47 (d, *J* = 4.8 Hz, 1H, H-6), 7.06 (d, *J* = 4.8 Hz, 1H, H-5), 7.20-7.30 (m, 5H, Ph), 4.16 (s, 2H, H-7), 3.69-3.71 (m, 4H, H-13 y H-13’), 2.93-2.94 (m, 4H, H-12 y H-12’), 2.58 (s, 3H, H-15). **^13^C NMR**  **(125 MHz, CDCl_3_) 10a**: δ = 203.2, 161.0, 147.8, 145.8, 139.7, 130.9, 128.6, 128.4, 126.2, 118.9, 67.4, 51.5, 40.5, 30.7. **^1^H NMR** **(500 MHz, CDCl_3_) 10'a**: δ = 8.89 (d, *J* =1.9 Hz, 1H, H-6’), 7.97 (d, *J* =1.9 Hz, 1H, H-4’), 7.20-7.30 (m, 5H, Ph), 4.33 (s, 2H, H-7’), 3.84-3.86 (m, 4H, H-13’), 2.89-2.90 (m, 4H, H-12’), 2.62 (s, 3H-15’). **^13^C NMR** **(125 MHz, CDCl_3_)** **10’a**: δ = 196.6, 162.0, 147.5, 145.1, 139.2, 131.3, 128.9, 128.4, 126.7, 126.3, 67.1, 52.7, 39.8, 26.8. **HRMS** of **10a** and **10’a**: C_18_H_20_N_2_O_2_ [M^+^] calc. 296.1520, found 296.1520. **FT-IR** (KBr) ν_max._ (cm^-1^) of **10a** and **10’a**: 2914.7-2850.8 (=C-H), 1695.6 (C=O), 1579.43 (C=N), 1407.86 (CH_3_-C=O).

Synthesis of ethyl 2-benzyl-3-morpholinylisonicotinate (**10c**) and ethyl 2-benzyl-3-morpholinylpyridine-5-carboxylate (**10'c**)

In a 50 mL round-bottom flask equipped with a magnetic stirrer, 4-benzyl-5-(4-morpholinyl)oxazole **4** (0.07 g, 0.27 mmol) was dissolved in CHCl_3_ (4 mL). After that, the dienophile **7c** (0.04 g, 0.27 mmol) was added. The resulting solution was stirred for 4 days at room temperature. After the reaction was complete, the solvent was evaporated under vacuum. NMR analysis of the crude reaction mixture revealed a 1:5 ratio of isomers **10c** and **10'c**. The mixture was purified by column chromatography using silica gel with Hex:AcOEt 8:1 (v/v) as the eluent, allowing the isolation of compound **10'c**. The yield of the pure mixture was 80%.

**^1^H NMR** **(500 MHz, CDCl_3_)** **10c**: δ = 8.43 (d, *J* = 4.8 Hz, 1H, H-6), 7.23 (d, *J* = 4.8 Hz, 1H, H-5), 7.15-7.30 (m, 5H, Ph), 4.41 (q, *J* = 7.14 Hz, 2H, H-15), 4.33 (s, 2H, H-7), 3.67-3.70 (m, 4H, H-13 y H-13’), 2.98-3.00 (bs, 4H, H-12 y H-12’), 1.4 (t, *J* = 7.14 Hz, 3H, H-16). **^1^H NMR** **(500 MHz, CDCl_3_) 10’c**: δ = 8.94 (d, *J* =1.9 Hz, 1H, H-6’), 8.00 (d, *J* =1.9 Hz, 1H, H-4’), 7.3 (m, 4H, Ph), 7.15-7.27 (m, 1H, H-11’), 4.39 (q, *J* = 7.13 Hz, 2H, H-15’), 4.31 (s, 2H, H-7’), 3.81-3.83 (m, 4H, H-13’ and H-13’), 2.85-2.87 (m, 4H, H-12’ y H-12’), 1.4 (t, *J* = 7.14 Hz, 3H, H-16’). **^13^C NMR** **(125 MHz, CDCl_3_)** **10’c**: δ = 165.46 (C-14’), 161.65 (C-2’), 147.06 (C-3’), 145.82 (C-6’), 139.30 (C-8’), 128.92 (C-4’), 128.87 (2C-10’), 128.37 (2C-9’), 126.26 (C-11’), 125.02 (C-5’), 67.14 (2C-13’), 61.35 (C-15’), 52.76 (2C-12’), 39.80 (C-7’), 14.32 (C-16’). **^15^N NMR (50.66 MHz, CDCl_3_)** **10’c**: δ = 317.8 (*N*-Pyridine,), 50.0 (*N*-Morpholine). **HRMS** of **10’c**: C_19_H_22_N_2_O_3_ [M^+^] calcd. 326.1630, found 326.1630. **FT-IR** (KBr) ν_max._ (cm^-1^) **10’c**: 2961.5-2853.7 (C-H), 1721.4 (C=O), 1590.3 (N=C), 1413.2 (CH_3_-C=O).

Synthesis of 2-benzyl-3-morpholinylpyridine (**10d**) and 2-benzyl-3-morpholinylpyridine-5-carboxylic Acid (**10'b**)

In a 50 mL round-bottom flask equipped with a magnetic stirrer, 4-benzyl-5-(4-morpholinyl)oxazole **4** (0.07 g, 0.27 mmol) was dissolved in CHCl_3_:DMSO (3:1 mL), followed by the addition of dienophile **7b** (0.03 g, 0.27 mmol). The solution was stirred for 7 days at room temperature. After the reaction was complete, the solvents were evaporated. NMR analysis of the crude reaction mixture revealed a 3:10 ratio of isomers para and meta **10b** and **10'b**, respectively. The product was purified by column chromatography using silica gel with Hex:AcOEt 5:1 (v/v) to obtain the less polar pyridine derivative **10d**. Then, pure ethyl acetate was used to isolate **10'b** as the major product. The yield of the pure mixture was 87%.

**^1^H NMR** **(500 MHz, CDCl_3_) 10d**: δ = 8.33 (dd, ^1^*J*= 1.5 y *^2^J* = 4.7 Hz, 1H, H-6), 7.42 (d, ^1^*J* = 1.5 y *^2^J* = 8.0 Hz, 1H, H-5), 7.22-7.28 (m, 5H, Ph), 7.15 (dd, ^1^*J* =4.7 Hz y *^2^J* =8.0 Hz, 1H, H-4), 4.27 (s, 2H, H-7), 3.79-3.81 (m, 4H, H-13 y H-13’), 2.79-2.81 (m, 4H, H-12 y H-12’). **^13^C NMR** **(125 MHz, CDCl_3_)** **10d**: δ = 157.2 (C-2), 147.2 (C-3), 144.8 (C-6), 140.1 (C-8), 128.8 (C-10 y C-10’), 128.4 (C-11), 128.2 (C-9 y C-9’), 125.9 (C-4), 122.1 (C-5), 67.2 (C-13 y C-13’), 52.8 (C-12 y C-12’), 39.6 (C-7). **HRMS** of **10d**: C_16_H_18_N_2_O [M^+^] calcd. 254.1419, found 254.1418. **FT-IR** (KBr) ν_max._ (cm^-1^) **10d**: 3056.9-2855.1 (C-H), 1574.4 (N=C), 1114.5 (CH_2_-O-CH_2_). **^1^H NMR** **(500 MHz, CDCl_3_) 10’b**: δ = 8.81 (d, *J* =1.9 Hz, 1H, H-6), 7.99 (d, *J* =1.9 Hz, 1H, H-4), 7.24-7.30 (m, 4H, Ph), 7.17-7.200 (m, 1H, H-11), 4.39 (s, 2H, H-7), 3.81-3.81 (m, 4H, H-13 and H-13’), 2.87-2.88 (m, 4H, H-12 y H-12’). **^13^C NMR** **(125 MHz, CDCl_3_) 10’b**: δ = 167.01 (C-14’), 161.12 (C-6’), 147.03 (C-5’), 145.73 (C-8’), 139.48 (C-2’), 129.15 (C-4’), 128.86 (2C-10’) 128.31 (2C-9’), 126.18 (C-11’), 66.97 (2C-13’), 52.70 (2C-12’), 39.57 (C-7’). **HRMS** of **10’b**: C_17_H_18_N_2_O_3_ [M^+^] calcd. 298.1318, found. 298.1318. **FT-IR** (KBr) ν_max._ (cm^-1^) **10’b**: 3418.4 (OH), 2925.3-2853.2 (C-H), 1586.9 (N=C), 1116.4 (CH_2_-O-CH_2_).

**Protonation Studies of Diene 4 in IEDDA Cycloadditions.**

To evaluate the role of *in-situ* protonation in the inverse electron-demand Diels–Alder (IEDDA) cycloaddition, we performed reactions using dienophiles lacking the carboxylic acid group: methyl 3-methoxyacrylate (**7f**), diethyl maleate (**7g**), and diethyl fumarate (**7h**) (Scheme S2). The cycloadditions were carried out in sealed tubes under nitrogen, varying temperature, solvent, and reaction time. No reaction was observed under any conditions (Table S1), even at high temperatures. These results indicate that the carboxylic acid proton is crucial for the cycloaddition. Further investigations using ^1^H, ^13^C, and ^15^N NMR spectroscopy were conducted to confirm the protonation of heterodiene 4, supporting the proposed IEDDA mechanism.

**Scheme S2**. IEDDA cycloadditions without protonated dienophiles.

**Table S1**. Diels–Alder cycloadditions of diene **4** with different dienophiles **7f-h** lacking the carboxylic acid group.

| **Entry** | **Solvent** | **Time (days)** | **Temperature (°C)** | **Product** |
| --- | --- | --- | --- | --- |
| 1 | Et₂O | 5 | 45 | --- |
| 2 | CHCl₃ | 15 | 50 | --- |
| 3 | THF | 7 | 100–150 | --- |
| 4 | MeOH | 10 | 40 | --- |
| 5 | MeOH | 10 | 40 | --- |
| 6 | DFE | 15 | 200 | --- |

**Theoretical calculations.**

**Computational Details and Energy Data.**

Theoretical calculations were performed at the M06-2X/6-311G(d,p) level of theory using *GAUSSIAN09*. Geometry optimizations were carried out for compounds **10c** and **10’c** in CHCl_3_ (using the polarizable continuum model (PCM)). Frequency calculations confirmed that all optimized structures correspond to true minima on the potential energy surface. The calculated HOMO–LUMO gaps and the sum of electronic and zero-point energies (in eV and a.u., respectively) are summarized below:

**Table S2**. Calculated HOMO–LUMO gaps and total electronic energies for **10c** and **10’c** in CHCl_3_ (using the polarizable continuum model (PCM) with zero-point energy correction) at the M06-2X/6-311G(d,p) level of theory.

| **Compound** | **Medium** | **HOMO–LUMO gap (eV)** | **Sum of electronic and ZPE (a.u.)** |
| --- | --- | --- | --- |
| **10c** | CHCl_3_ | 6.9 | −1071.892506 |
| **10’** | CHCl_3_ | 7.3 | −1071.895703 |

The data indicate that **10’c** possesses a deeper potential energy minimum than **10c**, consistent with its greater experimental abundance (**10c**:**10’c** = 1:5). The larger HOMO–LUMO gap observed for **10’c** also suggests a higher thermodynamic stability, supporting its identification as the major product in the reaction.

**Calculated Molecular Cartesian Coordinates**

Energies and cartesian coordinates of the optimized geometries of the species **10c** and **10’c**. They were obtained using chloroform as solvent with the polarizable continuum model (PCM) at the M06-2X/6-311G(d,p) level of theory using Gaussian 09 program (revision B.01).

2-benzyl-3-morpholinylisonicotinate (**10c**)

Sum of electronic and zero-point Energies = -1071.892506 au

HOMO Energy = -0.28011 au, LUMO Energy = -0.02602 au

Charge = 0; Multiplicity = 1

| **Atom** | **x** | **y** | **z** | **Atom** | **x** | **y** | **z** |
| --- | --- | --- | --- | --- | --- | --- | --- |
| C | -1.20678200 | -2.71587900 | -1.41178900 | H | 4.41690100 | 1.20853900 | -1.29427500 |
| C | -1.24191900 | -1.19802000 | -1.47131100 | C | 3.80517200 | -1.44956900 | 1.44159700 |
| C | -0.98151200 | -1.19197800 | 0.96471000 | H | 1.89523500 | -1.74617000 | 0.48677200 |
| C | -0.95059600 | -2.71240300 | 0.90123800 | C | 5.00317600 | -0.74097000 | 1.42293000 |
| H | -0.17239400 | -3.07029400 | -1.52715500 | H | 6.15255900 | 0.77013700 | 0.41462400 |
| H | -2.28127400 | -0.84935200 | -1.48958000 | H | 3.62825700 | -2.19560200 | 2.20793900 |
| H | -0.31095400 | -0.83727700 | 1.75222100 | H | 5.76095000 | -0.93145400 | 2.17373100 |
| C | -0.19867200 | 0.72459700 | -0.38472300 | H | 1.44364900 | -0.87192700 | -1.79717100 |
| C | -0.97379100 | 1.76140700 | 0.14913000 | O | -1.73270100 | -3.18432200 | -0.18145900 |
| C | 1.02148400 | 1.08726700 | -0.99357100 | H | -1.82068700 | -3.14454400 | -2.20407100 |
| C | -0.49025200 | 3.06695200 | 0.11822200 | H | 0.08147600 | -3.07159000 | 0.78976500 |
| N | -0.52330100 | -0.65663700 | -0.31440800 | H | 1.11172900 | 4.31845600 | -0.58227300 |
| N | 1.44797700 | 2.34657500 | -1.07242500 | H | -1.06424400 | 3.86945900 | 0.56374600 |
| C | 0.71490400 | 3.31082200 | -0.51654800 | C | -2.32969400 | 1.57628000 | 0.76227300 |
| C | 1.97221000 | 0.04500600 | -1.54374400 | O | -2.68733100 | 2.12736300 | 1.76950300 |
| H | 2.42486100 | 0.45598200 | -2.44711100 | O | -3.10467400 | 0.77519600 | 0.03347300 |
| H | -1.37576700 | -3.13669400 | 1.81085900 | C | -4.39101400 | 0.43674900 | 0.59364500 |
| H | -2.00760000 | -0.89687500 | 1.21769500 | H | -5.03443400 | 1.31688900 | 0.54163700 |
| H | -0.75053100 | -0.85320100 | -2.38530000 | H | -4.25123800 | 0.17800800 | 1.64530000 |
| C | 3.04965800 | -0.25156500 | -0.52113400 | C | -4.92962100 | -0.72404200 | -0.21111900 |
| C | 4.24999200 | 0.45784100 | -0.52952500 | H | -4.25668200 | -1.58154400 | -0.14005000 |
| C | 2.83452900 | -1.20381000 | 0.47630500 | H | -5.90906000 | -1.01501600 | 0.17092700 |
| C | 5.22261700 | 0.21393000 | 0.43487800 | H | -5.03515500 | -0.44707000 | -1.26132300 |

Ethyl 2-benzyl-3-morpholinylpyridine-5-carboxylate (**10'c**)

Sum of electronic and zero-point Energies=-1071.895703 au

HOMO Energy = -0.29339 au, LUMO Energy = -0.02523 au

Charge = 0; Multiplicity = 1

| **Atom** | **x** | **y** | **z** | **Atom** | **x** | **y** | **z** |
| --- | --- | --- | --- | --- | --- | --- | --- |
| C | -0.59433700 | 4.11502900 | 1.16557100 | H | 3.59492100 | -0.35902300 | 2.13909200 |
| C | -0.81421300 | 2.61121800 | 1.20540800 | C | 5.16417700 | -1.48932800 | -1.20265000 |
| C | -0.81375600 | 2.61125700 | -1.20530600 | H | 3.59565800 | -0.35775400 | -2.13911000 |
| C | -0.59391100 | 4.11507000 | -1.16534500 | C | 5.73204000 | -1.89832300 | -0.00009800 |
| H | 0.48331200 | 4.32727500 | 1.18890800 | H | 5.60033000 | -1.80396800 | 2.14357500 |
| H | -1.89515800 | 2.40807800 | 1.27500500 | H | 5.60108500 | -1.80267800 | -2.14375700 |
| H | -0.32482100 | 2.18405100 | -2.08417100 | H | 6.61273900 | -2.52970300 | -0.00013300 |
| C | -0.28200400 | 0.58624100 | 0.00010100 | H | 2.24259000 | 1.28159400 | -0.86964400 |
| C | -1.47825200 | -0.11441200 | 0.00008200 | O | -1.16939000 | 4.67762300 | 0.00001800 |
| C | 0.92358800 | -0.13930300 | 0.00005100 | H | -1.07246200 | 4.59600700 | 2.01881300 |
| C | -1.44476500 | -1.50682100 | 0.00004400 | H | 0.48374300 | 4.32733200 | -1.18829100 |
| N | -0.23007400 | 2.01476800 | 0.00015200 | H | -0.15373800 | -3.21741400 | -0.00002600 |
| N | 0.95077300 | -1.47208600 | 0.00000200 | H | -2.42801200 | 0.40861600 | 0.00010400 |
| C | -0.20425400 | -2.13354200 | 0.00000900 | C | -2.67303700 | -2.34717800 | 0.00004400 |
| C | 2.23738800 | 0.61781700 | 0.00005400 | O | -2.66630500 | -3.55195100 | -0.00003900 |
| H | 2.24260900 | 1.28151700 | 0.86981200 | O | -3.78820200 | -1.61351700 | -0.00005700 |
| H | -1.07174000 | 4.59606300 | -2.01874400 | C | -5.02748800 | -2.34926200 | -0.00013300 |
| H | -1.89467100 | 2.40809600 | -1.27531300 | H | -5.04941900 | -2.98949000 | 0.88358400 |
| H | -0.32561000 | 2.18398400 | 2.08444300 | H | -5.04926400 | -2.98955000 | -0.88381100 |
| C | 3.46058600 | -0.25980100 | -0.00000400 | C | -6.15047500 | -1.33946900 | -0.00026400 |
| C | 4.03581300 | -0.67633600 | 1.19947800 | H | -6.09911900 | -0.70670900 | -0.88731300 |
| C | 4.03621900 | -0.67563000 | -1.19953000 | H | -7.10987200 | -1.85878600 | -0.00033000 |
| C | 5.16375700 | -1.49004300 | 1.20250200 | H | -6.09927400 | -0.70665100 | 0.88675200 |


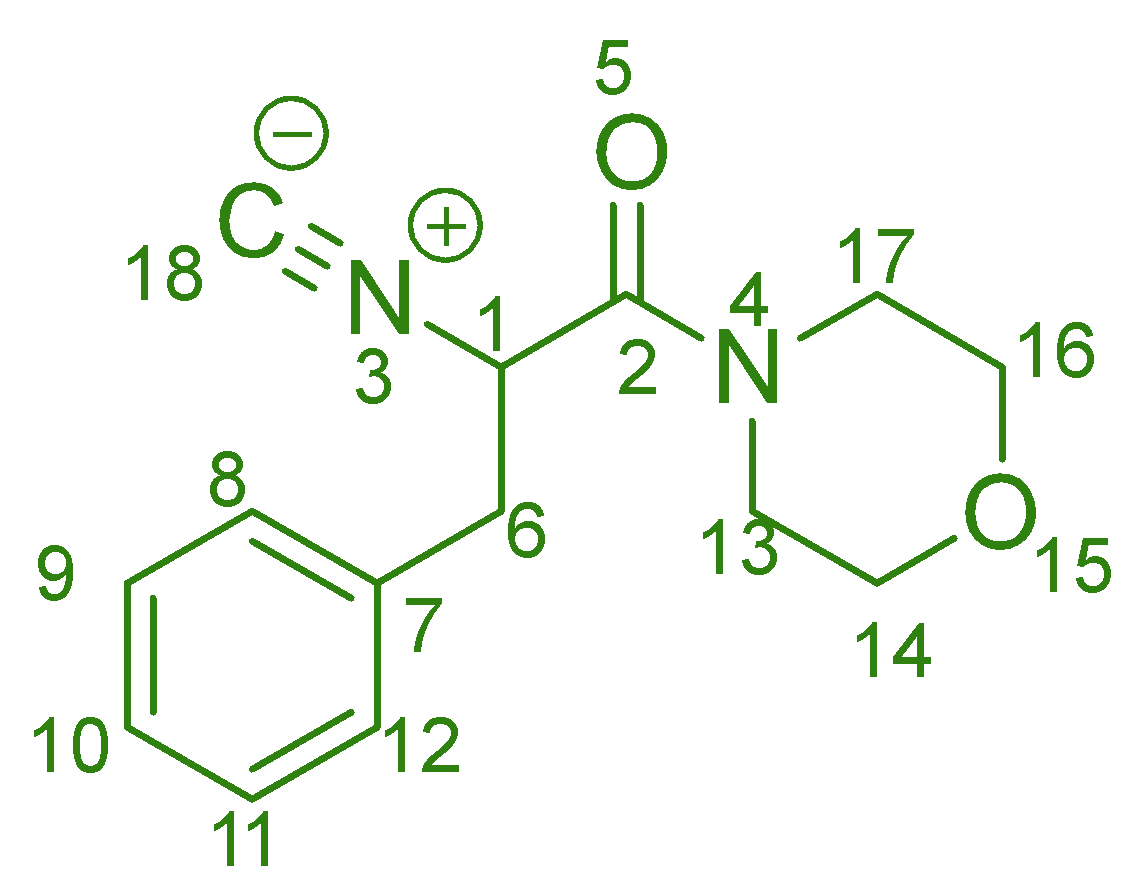


**Figure S1**. ^1^H NMR spectrum of 2-Isocyano-1-morpholino-3-phenylpropan-1-one (**3**) in CDCl_3_ at 500 MHz.


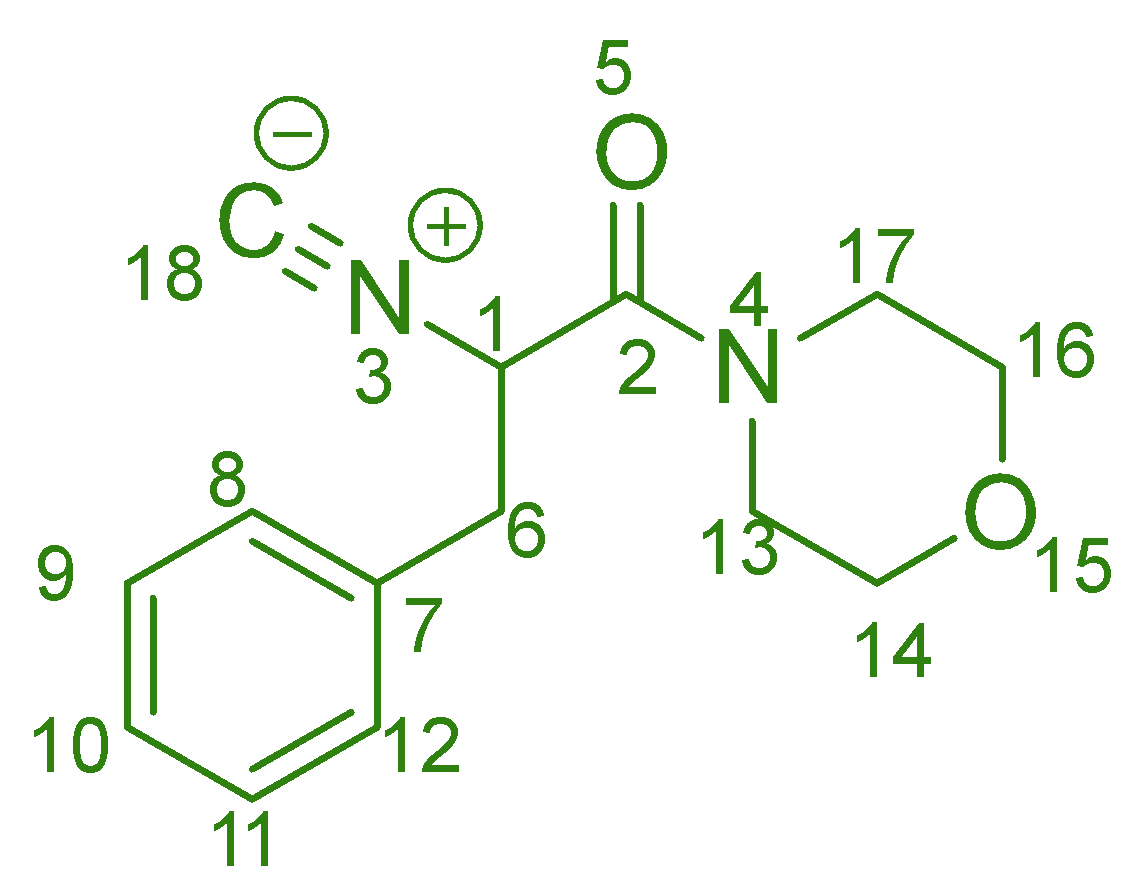


**Figure S2**. ^13^C NMR spectrum of 2-Isocyano-1-morpholino-3-phenylpropan-1-one (**3**) in CDCl_3_ at 126 MHz.

**

**Figure S3**. ^1^H NMR spectrum of 2-Isocyano-1-morpholino-3-phenylpropan-1-one (**3**) in CDCl_3_ at 500 MHz.

**Figure S4**. ^1^H NMR spectrum (500 MHz, CDCl_3_) of the diastereomeric mixture of **10a** and **10’a** that only shows the ^1^H signals of **10a**.

**Figure S5**. ^13^C NMR spectrum (125 MHz, CDCl_3_) of the diastereomeric mixture of **10a** and **10’a** that only shows the ^13^C signals of **10a**.

**Figure S6**. ^1^H NMR spectrum (500 MHz, CDCl_3_) of the diastereomeric mixture of **10a** and **10’a** that only shows the ^1^H signals of **10’a**.

**Figure S7**. ^13^C NMR spectrum (125 MHz, CDCl_3_) of the diastereomeric mixture of **10a** and **10’a** that only shows the ^13^C signals of **10’a**.

**Figure S8**. ^1^H NMR spectrum (500 MHz, CDCl_3_) of the diastereomeric mixture of **10c** and **10’c** that only shows the ^1^H signals of **10’c**.

**Figure S9**. ^13^C NMR spectrum (125 MHz, CDCl_3_) of the diastereomeric mixture of **10c** and **10’c** that only shows the ^13^C signals of **10’c**.

**Figure S10**. ^1^H NMR spectrum (500 MHz, CDCl_3_) of **10d**.

**Figure S11**. ^13^C NMR spectrum (125 MHz, CDCl_3_) of **10d**.

**Figure S12**. ^1^H NMR spectrum (500 MHz, CDCl_3_) of **10’b**.

**Figure S13**. ^13^C NMR spectrum (125 MHz, CDCl_3_) of **10’b**.

**^1^H NMR Analysis of Proton Transfer During the Cycloaddition**


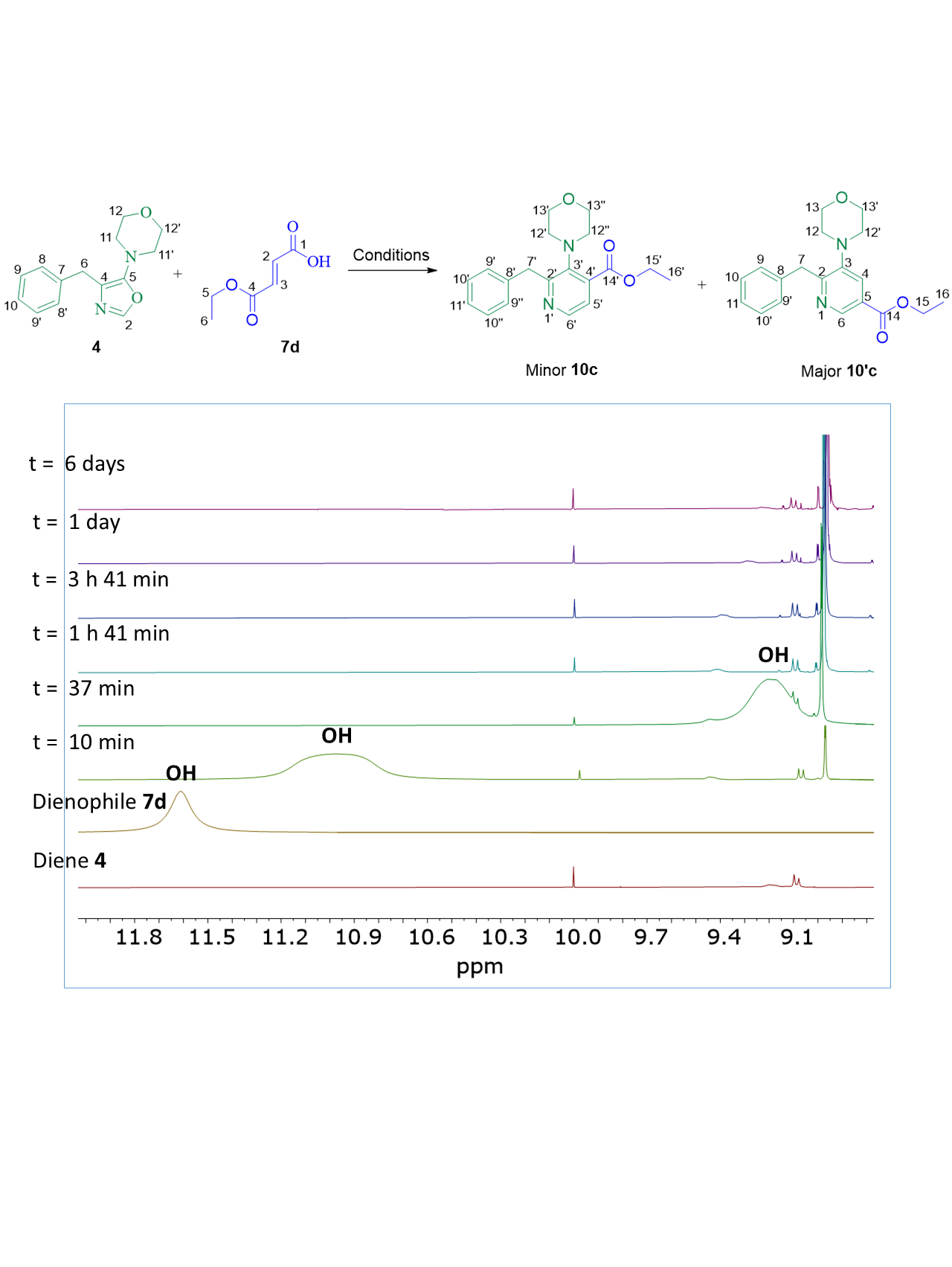


**Scheme S3**. Expanded region of the ^1^H NMR spectrum (500 MHz, CDCl_3_) illustrating the kinetics of the Diels–Alder cycloaddition between diene **4** and dienophile **7d**. Only the 9–12 ppm region is shown to highlight the proton transfer from **7d** to **4**.

**NMR experiments at different dienophile concentrations**

**Table S3.** Chemical shifts (δ, ppm) from ^1^H NMR (500 MHz, CDCl_3_) of compound **7d** at different concentrations.

| **Sample** | **Concentration⁽ᵃ⁾ [M]** | **H-1 (-COOH)** | **H-3** | **H-4** | **H-6** | **H-7** |
| --- | --- | --- | --- | --- | --- | --- |
| (a) | 0.541 | 11.62 | 6.95 | 6.85 | 4.29 | 1.33 |
| (b) | 0.271 | 11.22 | 6.95 | 6.85 | 4.29 | 1.33 |
| (c) | 0.136 | 10.62 | 6.95 | 6.85 | 4.29 | 1.33 |

⁽ᵃ⁾ Concentration [M] was calculated using the formula: [M]=nV=molL[M]

The results presented in Table S3 show a downfield chemical shift of 11.62 ppm for proton H-1 (-COOH) when the sample is concentrated. As the sample is progressively diluted, the chemical shift of H-1 moves upfield, reaching a δ value of 10.58 ppm. This shift can be attributed to the presence of intermolecular hydrogen bonds between the carboxylic acid proton of one molecule and another when the sample is in a concentrated state (Figure S14).

**Figure S14**. Intermolecular hydrogen bonding in compound **7d**.

It is well established in the literature that the chemical shift of a hydrogen atom involved in hydrogen bonding moves downfield (higher frequency) due to the sharing of its electron density with two electronegative sites.^5^ Therefore, as the sample becomes more diluted, the probability of interaction with neighboring molecules decreases, causing the chemical shift to move upfield (lower frequency).

The vinyl protons, on the other hand, exhibited doublets for H-3 and H-4 with a coupling constant of approximately 15.77 Hz when the sample was concentrated. However, no significant changes were observed for these protons, nor for the methylene (-CH₂-, H-6) and methyl (-CH₃, H-7) groups (see Figure S15).


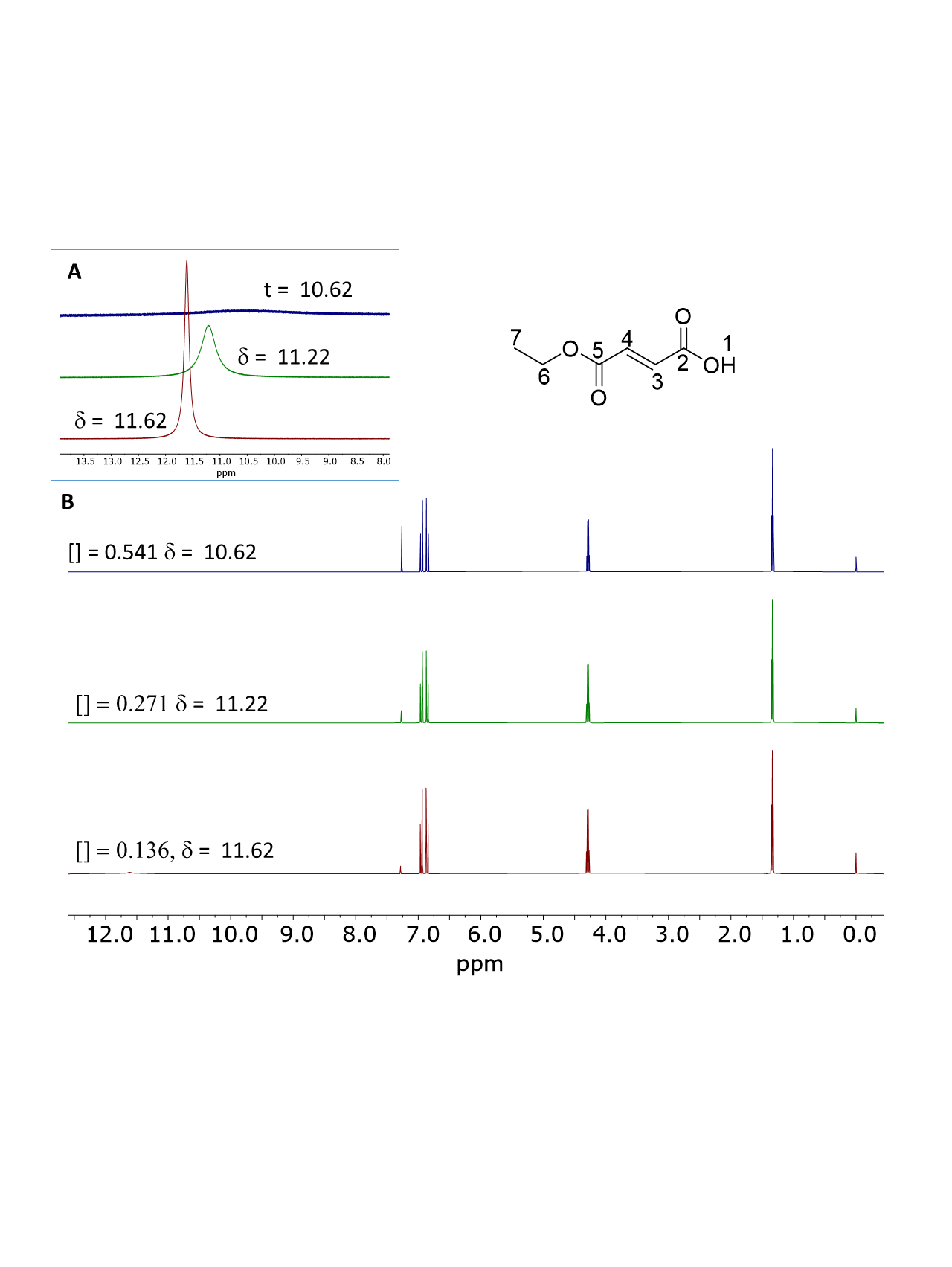


**Figure S15**. ^1^H NMR spectrum (500 MHz, CDCl_3_) of acid **7d**.

Analysis of the ^13^C NMR spectra revealed no variation in chemical shifts or carbon signals across different concentrations: 170.13 ppm for C-2, 135.67 ppm for C-3, 132.47 ppm for C-4, 164.58 ppm for C-5, 61.56 ppm for C-6, and 14.04 ppm for C-7 (Figure S16).


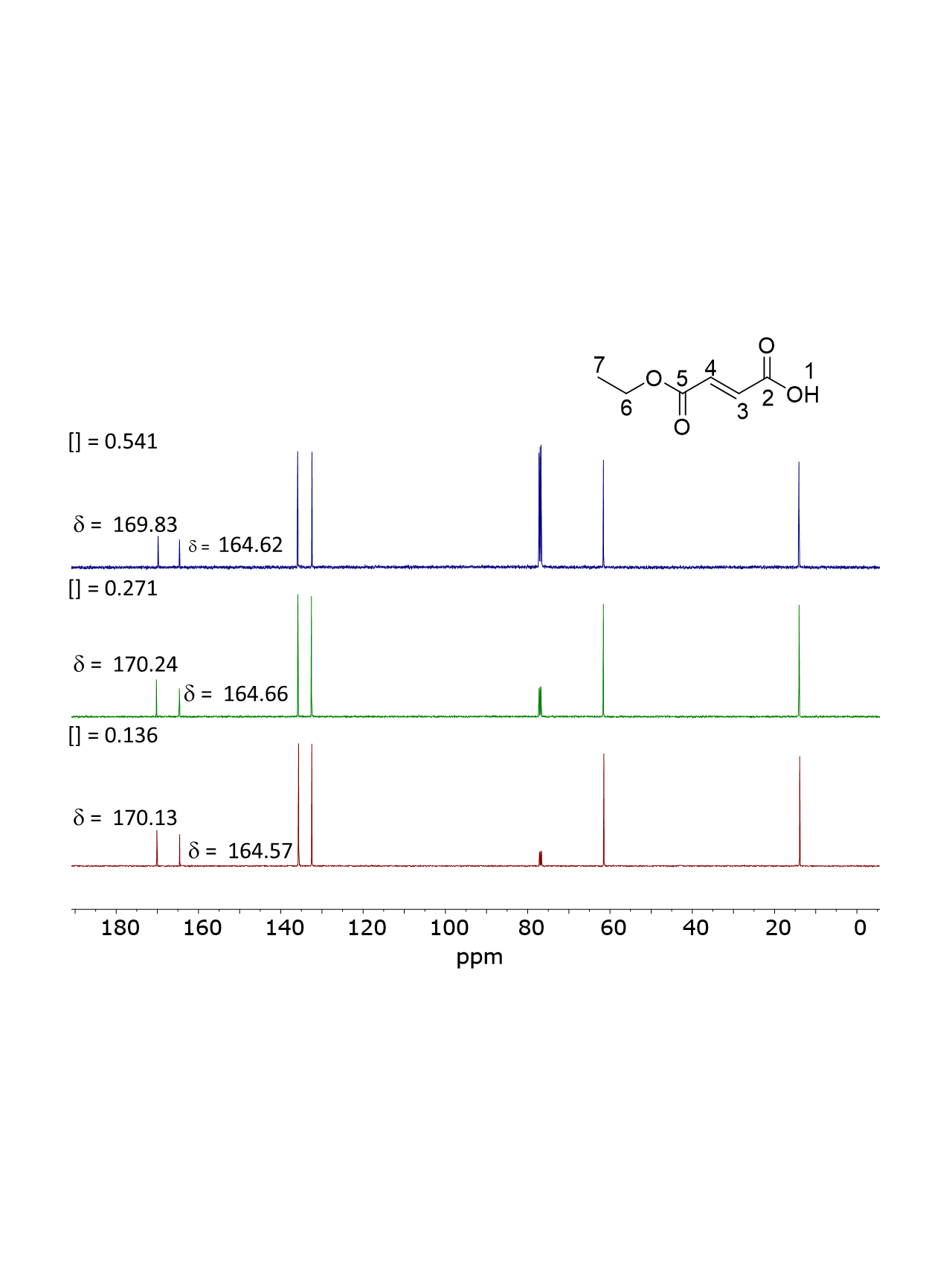


**Figure S16.** ^13^C NMR spectrum (125 MHz, CDCl_3_) of acid **7d**.

This study confirms that, regardless of concentration, the doublet signals corresponding to vinyl protons H-3 and H-4 remain unchanged. Consequently, we can rule out that the observed chemical shift variations arise from concentration effects in the reaction between compound **4** and **7d**. Following this analysis, we proceeded to investigate protonation, hydrogen bonding interactions, and potential π–π stacking interactions.

**Protonation or N–H···O Hydrogen Bonding Interaction vs π–π Stacking Interaction**

Given the π-electron-rich nature of the systems under study, it was important to determine whether the observed chemical behavior during the reaction arose from π–π stacking interactions between the diene **4a** and the dienophile **7d**, from a potential N–H···O hydrogen bonding interaction, or from protonation of the nitrogen atom in the diene **4a**.

To evaluate these possibilities and deepen our understanding of the Diels–Alder cycloaddition between **4** and **7d**, we conducted NMR studies on diene **4a** and dienophile **7d** under identical reaction conditions. The goal was to assess whether the cycloaddition proceeded via in situ protonation of the nitrogen atom and to observe the corresponding chemical shift changes in both diene and dienophile for comparison purposes. The results of this investigation are presented below.

**^1^H NMR Spectroscopy**

Upon analyzing and comparing the ^1^H NMR spectra of isolated diene **4a** and dienophile **7d** with those obtained during the reaction, we observed a slight downfield shift of proton H-1 (–COOH, dienophile) and H-2 (–O–CH–N, diene), suggesting possible protonation of the nitrogen atom or the formation of a hydrogen bond (N–H···O). Notably, a similar trend was observed in the Diels–Alder reaction between 4-benzyl-5-(4-morpholinyl)oxazole **4** and **7d**, where the doublets corresponding to H-3 and H-4 exhibited altered splitting patterns due to the presence of the oxazole ring. In the case of **4a**, the AB system of first-order transitions transformed into a nearly magnetically equivalent second-order system (Figure S17).


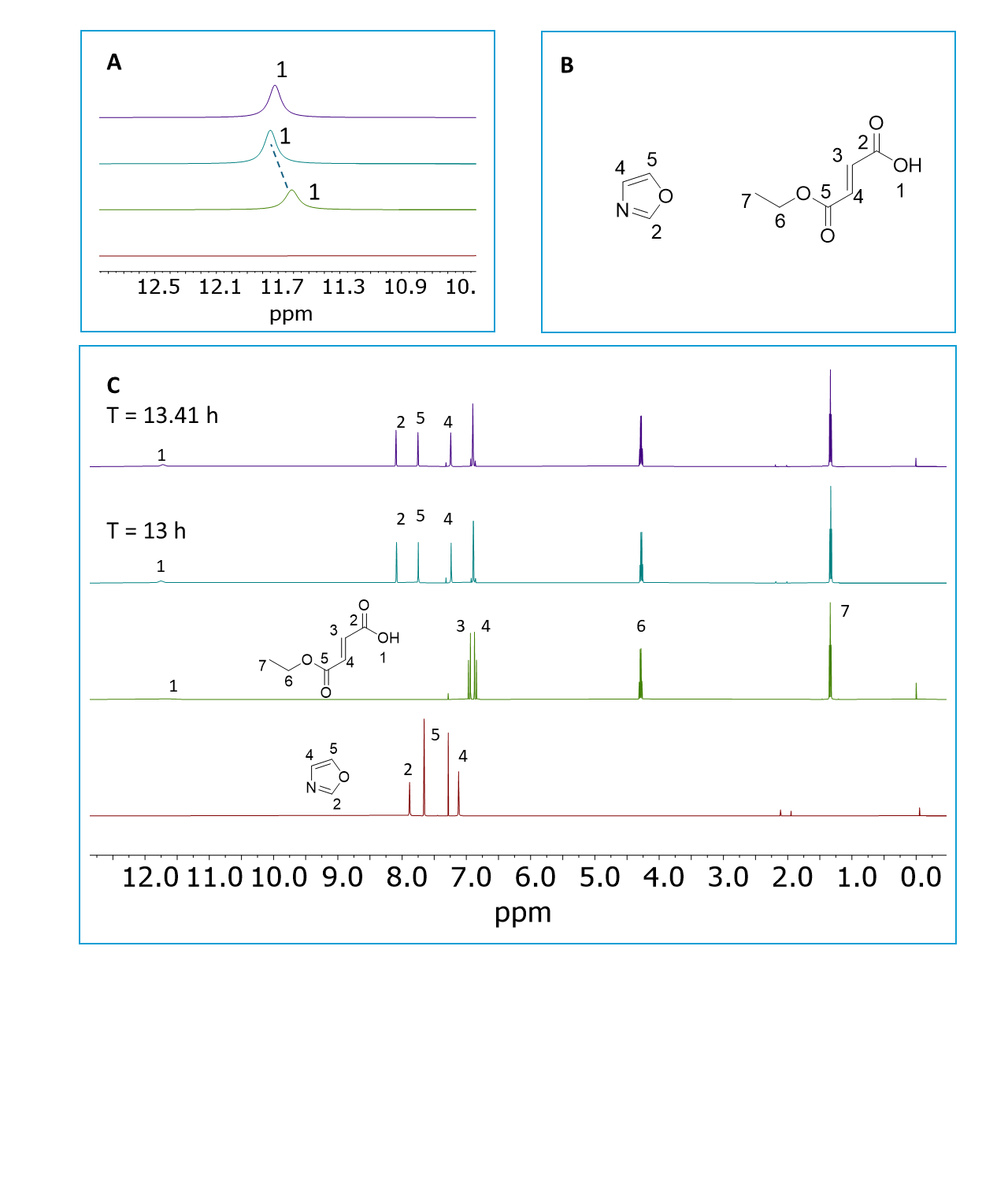


**Figure S17**. ^1^H NMR spectrum (500 MHz, CDCl_3_) of the cycloaddition reaction between diene **4a** and dienophile **7d**. A) Expanded region from 10 to 12 ppm highlighting the chemical shift of the acidic proton H-1. B) Chemical structures of the diene and dienophile and (C) cycloaddition reaction between **4a** and **7d**, with ¹H NMR spectra recorded at T = 13 h (blue) and T = 13.41 h (purple).


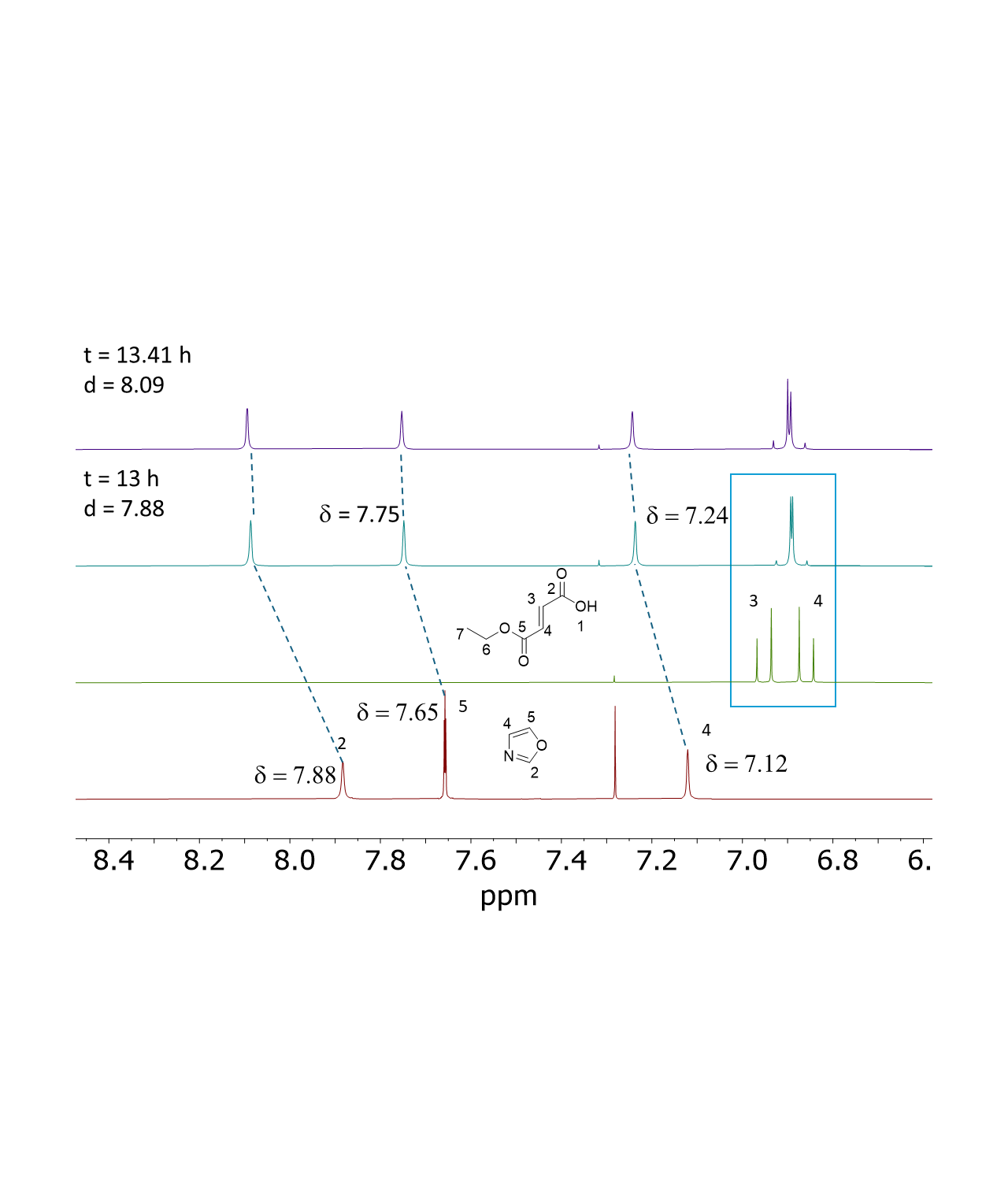


**Figure S18**. Magnification of the ^1^H NMR spectrum (500 MHz, CDCl_3_) showing chemical shift changes in ppm.

**^13^C NMR Spectroscopy**

The ^13^C NMR spectra further supported these observations. Similar to the ^1^H NMR results of the reaction between **4** and **7d**, the ^13^C NMR spectra revealed analogous shielding and deshielding effects for the carbon atoms in dienophile **7d**. The carboxylic acid carbon (C-2) exhibited an upfield shift of approximately 2 ppm, while C-5 showed a slight downfield shift. The vinyl carbons also experienced downfield shifts of about 1 ppm each. These chemical shift changes closely resemble those observed for **7d** in the presence of diene **4**, prompting further investigation through ^15^N NMR spectroscopy.


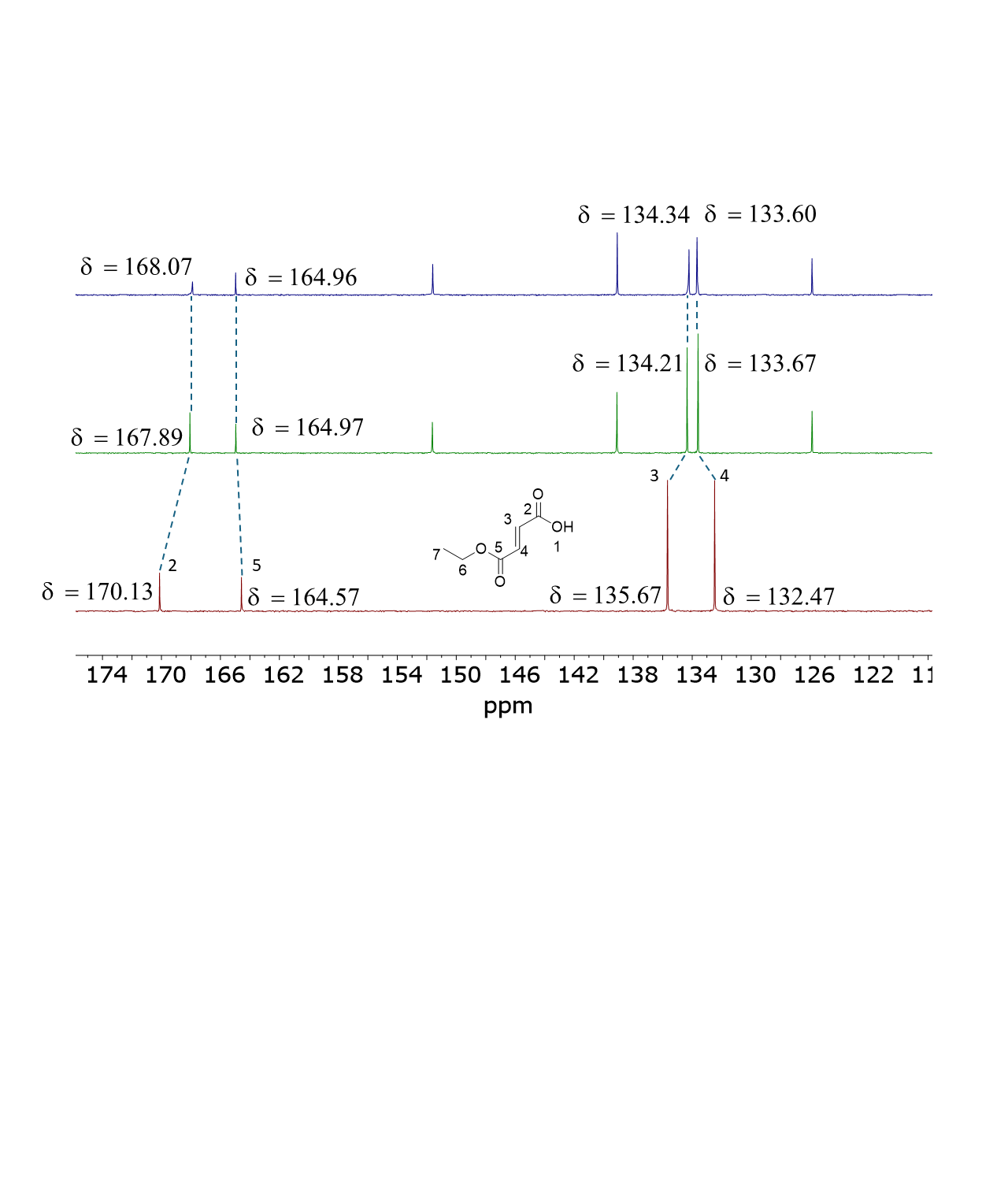


**Figure S19**. Magnification of ^13^C NMR spectrum (125 MHz, CDCl_3_) showing chemical shift changes in ppm.

**^15^N NMR Spectroscopy**


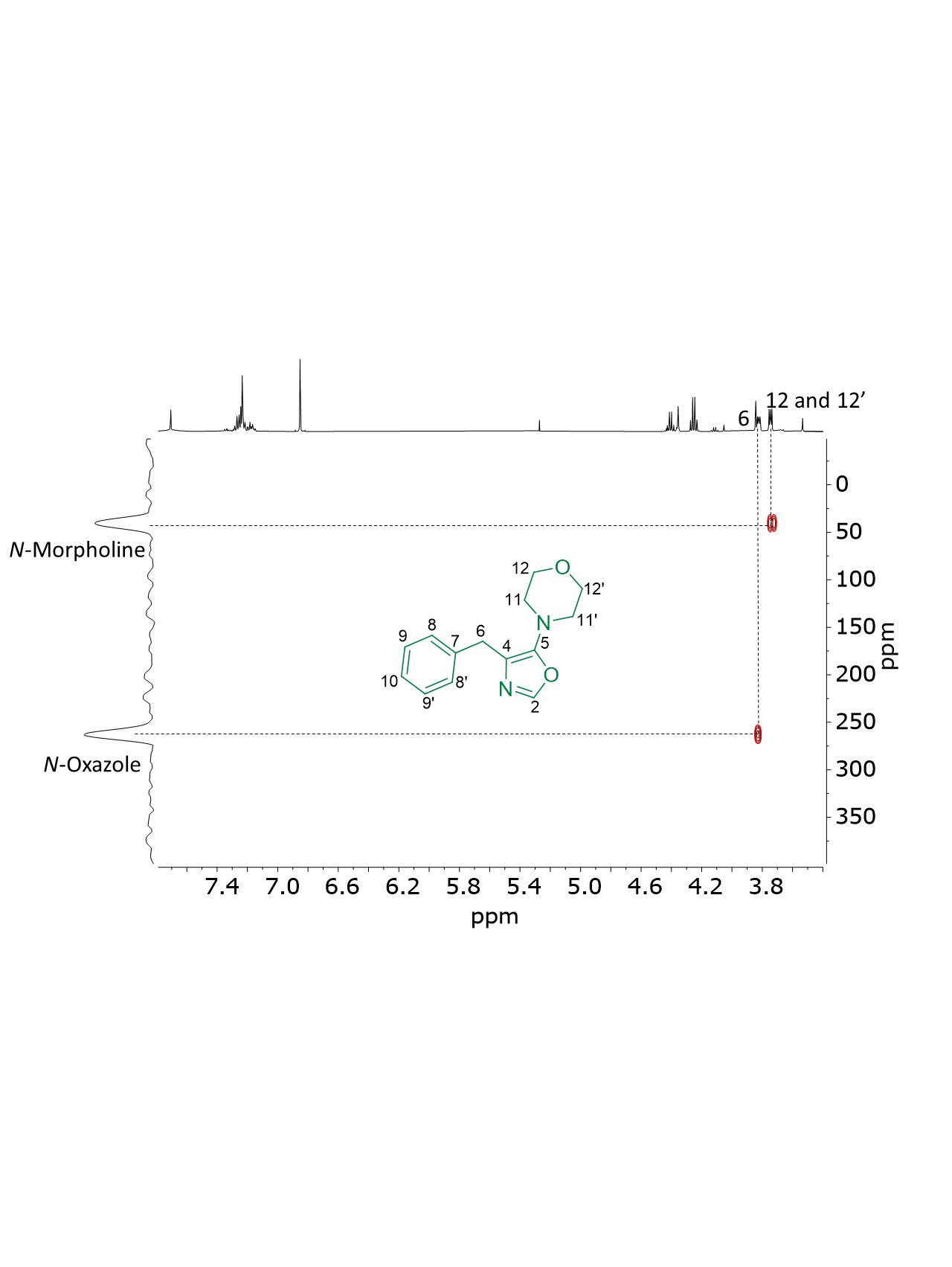


**Figure S20.** ^15^N NMR spectrum (50.66 MHz, *J^3^*_1H,15N_= 3 Hz, CDCl_3_) of oxazole **4**, using ammonia as a reference.

Comparison of the ^15^N chemical shifts of starting materials **4a** and **4** with their respective mixtures with dienophile **7d** revealed similar behavior in both reactions. Initially, the isolated dienes showed higher-frequency ^15^N signals, which shifted downfield upon interaction with **7d**, indicating a change in the nitrogen environment (see Table S4).

**Table S4**. ^15^N NMR chemical shifts (δ, ppm; 50.66 MHz, CDCl_3_).

| Compound | δ ^15^N (ppm) |
| --- | --- |
| **4a** (oxazole) | 254.30 |
| **4a** + **7d** | 249.71 |
| **4** (4-benzyl-5-(4-morpholinyl)oxazole) | 262.48 |
| **4** + **7d** | 255.89 |

These values were compared with literature-reported chemical shifts for protonated species (δ ^15^N = 190–250 ppm). Despite the chemical shift trends, the cycloaddition between **4a** and **7d** did not proceed even after 7 days, leading us to propose the formation of a non-covalent N–H···O hydrogen bond between the reactants rather than full protonation. However, considering the π-rich nature of both components, the possibility of a π–π stacking interaction was also plausible. To distinguish between these alternatives and better understand the nature of the interaction, additional reactions were designed and conducted.

**References**

1. Annangudi, S. P., Sun, M., & Salomon, R. G. (2005). An Efficient Synthesis of 4-Oxoalkenoic Acids from 2-Alkylfurans. Synlett, 9, 1468–1470. <https://doi.org/10.1055/s-2005-869833>
2. Bustos, C., Salgado, G., Martínez, R., & Carrière, F. (2000). Determinacion de la configuracion E-Z de los acidos Fumarico y Maleico. Un experimento orientado a incentivar el desarrollo de la investigacion cientifica en alumnos de Pregrado. Química Nova, 23(4), 568–570. <https://doi.org/10.1590/S0100-40422000000400023>
3. Ekici, Ö. D., Li, Z. Z., Campbell, A. J., James, K. E., Asgian, J. L., Mikolajczyk, J., Salvesen, G. S., Ganesan, R., Jelakovic, S., Grütter, M. G., & Powers, J. C. (2006). Design, Synthesis, and Evaluation of Aza-Peptide Michael Acceptors as Selective and Potent Inhibitors of Caspases-2, -3, -6, -7, -8, -9, and -10. Journal of Medicinal Chemistry, 49(19), 5728–5749. <https://doi.org/10.1021/jm0601405>
4. M. J. Frisch, G. W. T., H. B. Schlegel, G. E. Scuseria, M. A. Robb, J. R. Cheeseman, G. Scalmani, V. Barone, B. Mennucci, G. A. Petersson, H. Nakatsuji, M. Caricato, X. Li, H. P. Hratchian, A. F. Izmaylov, J. Bloino, G. Zheng, J. L. Sonnenberg, M. Hada, M. Ehara, K. Toyota, R. Fukuda, J. Hasegawa, M. Ishida, T. Nakajima, Y. Honda, O. Kitao, H. Nakai, T. Vreven, J. A. Montgomery, Jr., J. E. Peralta, F. Ogliaro, M. Bearpark, J. J. Heyd, E. Brothers, K. N. Kudin, V. N. Staroverov, T. Keith, R. Kobayashi, J. Normand, K. Raghavachari, A. Rendell, J. C. Burant, S. S. Iyengar, J. Tomasi, M. Cossi, N. Rega, J. M. Millam, M. Klene, J. E. Knox, J. B. Cross, V. Bakken, C. Adamo, J. Jaramillo, R. Gomperts, R. E. Stratmann, O. Yazyev, A. J. Austin, R. Cammi, C. Pomelli, J. W. Ochterski, R. L. Martin, K. Morokuma, V. G. Zakrzewski, G. A. Voth, P. Salvador, J. J. Dannenberg, S. Dapprich, A. D. Daniels, O. Farkas, J. B. Foresman, J. V. Ortiz, J. Cioslowski, and D. J. Fox *Gaussian 09, Revision B.01*, 2010, Gaussian, Inc.: Wallingford CT.
5. H. Günter, *NMR Spectroscopy: Basic Principles, Concepts and Applications in Chemistry*, **2013**, 3rd ed., Wiley.
